# Substitutional Single Iron Atoms as Active Sites for Nitrophenol Reduction by the Mo_2_C MXene

**DOI:** 10.1021/acsanm.6c02370

**Published:** 2026-07-09

**Authors:** Sabine Eliane Midré, Victor Ramón-Trimiño, Antón López-Francés, Sergio Navalón, José D. Gouveia, José R. B. Gomes, Sara Goberna-Ferrón, Hermenegildo García, Ana Primo

**Affiliations:** † Instituto Universitario de Tecnología Química, Consejo Superior de Investigaciones Científicas, 16774 Universitat Politècnica de València, Universitat Politècnica de València, Av. De los Naranjos s/n, Valencia 46022, Spain; ‡ Departamento de Química, Universitat Politècnica de València, Av. De los Naranjos s/n, Valencia 46022, Spain; § CICECO–Aveiro Institute of Materials, Department of Physics, University of Aveiro, Campus Universitário de Santiago, Aveiro 3810-193, Portugal; ∥ CICECO–Aveiro Institute of Materials, Department of Chemistry, University of Aveiro, Campus Universitário de Santiago, Aveiro 3810-193, Portugal

**Keywords:** single atom catalyst, Mo_2_C MXene, nitrophenol, heterogeneous catalysts, DFT calculations

## Abstract

Etching of Mo_2_Ga_2_C using in situ generated HF to produce Mo_2_C MXene leads to the generation of atomic vacancies that can serve as anchoring sites for Fe single atoms. High-angle annular dark-field scanning transmission electron microscopy (HAAD-STEM) shows the absence of clusters or nanoparticles. Data from extended X-ray absorption fine structure (EXAFS) regarding Fe coordination fit well with the distances and environment calculated with density functional theory (DFT) for the substitution of Mo by Fe single atoms in the MXene lattice. Computational data also indicate that incorporation of Fe in the Mo layer favors a surface free of functional groups and ready to interact with substrates. These theoretical insights correlate with the experimentally observed high catalytic activity of Fe single atoms on Mo_2_C for nitroarene reduction by aqueous NaBH_4_, a benchmark reaction in organic synthesis for evaluating heterogeneous catalysts. Activity data show that Fe single atoms on Mo_2_C rank among the most active materials reported so far for this reaction.

## Graphical abstract

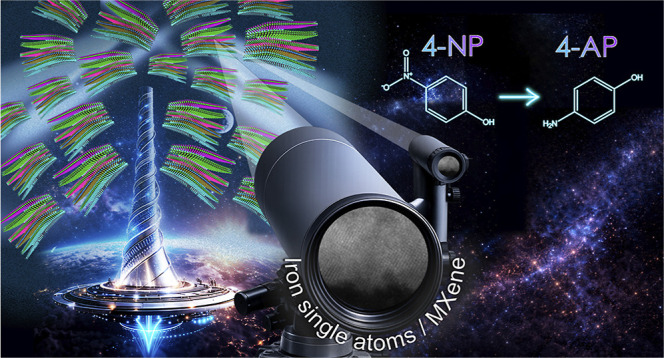

## Introduction

4-Nitrophenol (4-NP) is a highly persistent toxic pollutant frequently present in industrial waste waters.^1,2^ Widely released by chemical industries such as dye, petroleum, and pesticide manufacturing, it is classified by the U.S. Environmental Protection Agency as a priority pollutant.^3^ Interestingly, the reduction product of 4-NP, 4-aminophenol (4-AP), is far less toxic and has substantial industrial value. 4-AP serves as a critical intermediate in pharmaceuticals (e.g., as a precursor for paracetamol, vitamin B1, and analgesics), in dye manufacturing (direct, sulfur, and disperse dyes), and in applications spanning the rubber and photographic industries. Therefore, the selective catalytic conversion of 4-NP to 4-AP is an economically attractive route to high-value chemicals.^4,5^

Catalytic hydrogenation using NaBH4 and solid catalysts has been widely explored for this transformation. Noble metal nanoparticles (such as Pd, Pt, Au, and Ag) demonstrate excellent performance but remain limited by scarcity, high cost, and aggregation.^5^ Non-noble metals such as Cu,^6^ Ni,^7,8^ Co,^9^ and Fe^10^ are more abundant and cost-effective, but often suffer from lower activity or stability. To maximize metal utilization and stability, single-atom catalysts (SACs) have emerged as a new Frontier, offering unique electronic properties, enhanced selectivity, and higher stability due to the prevention of agglomeration.^11–13^

Stabilizing isolated atoms requires supports with strong anchoring capability and efficient electron transfer. MXenes, a family of two-dimensional transition metal carbides and nitrides, are particularly well-suited for this purpose.^14^ Their tunable surface chemistry, which modulates adsorption energy, the large percentage of accessible atoms due to the 2D morphology, and the presence of atom vacancies created during synthesis facilitate electron shuttling and dispersion of active species.^15–18^ Recent studies have demonstrated that MXene-supported composites containing Au,^19,20^ Ag,^21–23^ Cu,^24–26^ Bi,^27^ or Mo^28^ achieve rapid and complete reduction of 4-NP with high recyclability. However, most efforts remain focused on nanoparticles or clusters, and the potential of MXene-supported SACs for this reaction remains largely unexplored.

Herein, we report the synthesis and catalytic activity for NaBH_4_ hydrogenation of 4-NP of Fe single atoms (SA) anchored on few-layer Mo_2_C MXene (Fe­(SA)/Mo_2_C) as a highly efficient and cost-effective solid catalyst. This approach combines the advantages of atomic dispersion with the unique physicochemical properties of MXenes to achieve superfast, selective, and durable catalytic conversion of 4-NP to 4-AP.

## Materials and Methods

### Materials

Mo_2_Ga_2_C was supplied by Nanochemazone (ref NCZ-RS-110/25). HCl was supplied by Fisher Scientific (ref AC423790025). NH_4_F (ref 216011), Fe­(CO_2_CH_3_)_2_ (ref 339199), Fe_3_O_4_ (ref 637106), NaBH_4_ (ref 452882), 4-nitrophenol (ref 241326), 4-nitroaniline (ref 185310) and nitrobenzene (ref 252379), were all supplied by Sigma-Aldrich.

### Synthesis of Mo_2_C MXene

Commercial Mo_2_Ga_2_C was etched to Mo_2_C MXene through an in situ approach, designed to avoid direct handling of hydrofluoric acid (HF), thereby improving laboratory safety. To generate in situ HF, 1.5 g of ammonium fluoride (NH_4_F) was dissolved in 9 mL of ultrapure Milli-Q water and mixed with 15 mL of hydrochloric acid (HCl). The obtained mixture has a maximum concentration of 14 wt % HF. Subsequently, 0.5 g of Mo_2_Ga_2_C precursor was slowly added to the solution. The resulting suspension was heated to 65 °C and stirred continuously for 7 days. After the reaction, 480 mL of ultrapure Milli-Q water was added to further dilute the HF concentration to less than 1 wt %. Then, Mo_2_C was filtered, thoroughly washed with ultrapure Milli-Q water, and dried.

### Synthesis of Fe­(SA)/Mo_2_C

To synthesize Fe single atoms on the MXene, 100 mg of the previously obtained Mo_2_C were dispersed in 10 mL of Milli-Q water. Independently, 2.1 mg of iron acetate were dissolved in 10 mL of Milli-Q water to achieve Fe­(SA)/Mo_2_C with a weight percent of 0.6 of Fe. Additionally, catalysts with lower (0.3 wt %) and higher (1 wt %) Fe loadings were synthesized and analyzed alongside the 0.6 wt % Fe sample to enable comparison and evaluate the optimal Fe content for catalytic performance. For this we used 1.04 mg and 3.1 mg of iron actetate to create the impregnation dissolution. The Mo_2_C suspension was then slowly added to the iron acetate solution. The resulting suspension was heated to 80 °C with vigorous stirring for 20 h. Following the reaction, the material was filtered, washed and freeze-dried.

Additionally, for comparison, a physical mixture of Fe_3_O_4_ and MXene was prepared and analyzed to evaluate its behavior relative to the synthesized catalyst. This mixture was prepared by combining 200 mg of as-synthesized Mo_2_C with 1.66 mg of Fe_3_O_4_, followed by grinding with a mortar and pestle for 10 min to obtain a homogeneous mixture.

### Physicochemical Characterization

Elemental analysis was carried out using inductively coupled plasma-optical emission spectroscopy (ICP-OES) on a PerkinElmer Avio 560 Max instrument. Prior to analysis, the samples were digested using an acid mixture.

X-ray photoelectron spectroscopy (XPS) analyses were performed using a SPECS spectrometer equipped with a Phoibos 150MCD-9 detector using nonmonochromatic Mg Kα radiation (50W, 1253.6 eV) and a multichannel detector. The fresh Fe­(SA)/Mo_2_C sample (30 mg) was analyzed as a powder. In contrast, the post-mortem sample (7 mg) was dispersed in ethanol, drop-cast onto a quartz substrate, and measured without further treatment. XPS data were processed by using Casa XPS software, applying the Shirley function for background correction. The C 1s signal of adventitious carbon 284.8 eV was used as a reference for calibration.

X-ray Diffraction (XRD) patterns were acquired in Bragg–Brentano geometry using a PANalytical CUBIX diffractometer equipped with a PANalytical X’Celerator detector. X-ray radiation of Cu Kα (λ1 = 1.5406 Å, λ2 = 1.5444 Å, I2/I1 = 0.5) was used, with a tube voltage and intensity of 45 kV and 40 mA respectively. The length of the arm of the goniometer was 200 mm, and a slit of variable divergence with an irradiated sample area of 3 mm was used. The measurement range was from 2.0°to 90.0° (2θ), with a step of 0.020° (2θ). The measurement was carried out at 298 K, rotating the sample during it at 0.5 revolutions per second. Fresh samples were analyzed as powders with a counting time of 17 s per step. Due to their limited quantity, postreaction samples were dispersed in ethanol, drop-cast onto a zero-background sample holder, and measured with a longer counting time of 100 s per step to improve resolution.

Field-emission scanning electron microscopy (FESEM) images were obtained using a ZEISS GeminiSEM 500 microscope equipped with an energy-dispersive X-ray detector. Samples were mounted on conductive carbon tape. For high-resolution imaging, high annular dark-field scanning transmission electron microscopy (HAADF-STEM) and high-resolution transmission electron microscopy were performed. Samples were ultrasonically dispersed in ethanol and drop-cast onto 400-mesh copper grids. Imaging and elemental mapping were carried out using a Titan G2 60–300 aberration-corrected TEM operating at 300 kV. Additional TEM images were acquired using a Bruker system at an accelerating voltage of 200 kV. The thickness of MXene flakes was determined by atomic force microscopy (AFM) analysis with a Multimode Nanoscope 3 A instrument.

The X-ray absorption spectroscopy (XAS) (Fe K-edge) data were collected at BL14W beamline in Shanghai Synchrotron Radiation Facility (SSRF). The storage rings of SSRF were operated at 3.5 GeV with a stable current of 200 mA. Using Si(111) double-crystal monochromator, the data collection was carried out in fluorescence mode using Lytle detector. All spectra were collected in ambient conditions. X-ray absorption fine structure (XAFS) data were processed using the Athena module implemented in the IFEFFIT software packages. The Extended XAFS (EXAFS) spectra were obtained by subtracting the postedge background from the overall absorption and then normalizing with respect to the edge-jump step. Prior to fitting the sample data, a reference fit was performed using Fe foil and its corresponding CIF file to determine the amplitude reduction factor (Δ*S*_0_^2^) and energy shift (Δ*E*_0_), and these parameters were subsequently fixed and applied in the fitting of the studied materials. Subsequently, the χ­(*k*) data were Fourier transformed into real (*R*) space using a Hann window function (dk = 1.0 Å^–1^) to separate the EXAFS contributions from different coordination shells. To obtain the quantitative structural parameters around central atoms, least–squares curve parameter fitting was performed using the ARTEMIS module of IFEFFIT software package.^29,30^

### Catalytic Performance for the Reduction of 4-Nitrophenol

Reaction solutions were prepared by dispersing 8 mg of the catalyst in 11.5 mL of Milli-Q water containing 1 mL of a 40 mM solution of 4-nitrophenol (4-NP). The mixture was sonicated for 15 min to ensure the complete dissolution of the reactant and a homogeneous dispersion of the catalyst. Subsequently, the suspension was stirred for 30 min at 60 °C. Separately, 120 mg of NaBH_4_ were dissolved in 12.5 mL of Milli-Q water. Once fully dissolved, this solution was mixed with the catalyst suspension under continuous stirring. Aliquots were withdrawn at specific time intervals, passed through a 0.45 μm nylon filter, and diluted in a 1:25 ratio with Milli-Q water. The reaction progress was monitored via UV–Vis spectroscopy (200–500 nm range) by tracking the decrease of the characteristic absorbance band at 400 nm. Calibration curves for 4-nitrophenol (4-NP) and 4-aminophenol (4-AP) were obtained by UV–vis spectroscopy using standard solutions with known concentrations under identical experimental conditions (Figure S1). The absorbance values at the characteristic wavelengths of 4-NP and 4-AP were employed to construct the corresponding linear calibration plots, which were subsequently used to quantify substrate conversion and product formation during the catalytic reactions.

### EPR Spectroscopy Analysis

Liquid-phase electron paramagnetic resonance (EPR) experiments were conducted using DMPO as a spin-trap agent. An aqueous mixture (25 mL) was prepared containing DMPO (1 g·L^–1^), the catalyst (8 mg), and NaBH_4_ (120 mg). After 30 min of continuous stirring, the mixture was filtered through a 0.45 μm nylon membrane and deoxygenated by purging with argon for 20 min. EPR spectra were acquired using a Bruker EMS spectrometer with a microwave frequency of 9.803 GHz, sweep width of 3489.9 G, time constant of 40.95 ms, modulation frequency of 100 kHz, modulation amplitude of 1 G, and microwave power of 19.92 mW.

### Theoretical Calculations

The density functional theory (DFT)-based calculations were carried out using the VASP code.^31^ The calculations were carried out using the well-established PBE-D3 combination,^32,33^ which accounts for van der Waals interactions. This approach has demonstrated accuracy compared to experimental results^34–37^ in numerous first-principles computational studies of the interaction between the surfaces of MXenes and adsorbates,^38^ including some of our own.^39–43^

The explicitly treated electrons for each element were the valence ones, except in the case of Mo, for which a total of 14 electrons (4s^2^ 4p^6^ 4d^5^ 5s^1^) were explicitly considered, as recommended by the VASP documentation. The remaining electrons of each element were treated as the core with the projector augmented-wave method.^44^

A periodic supercell model containing 4 × 4 Mo_2_CO_2_ MXene unit cells (80 atoms) was used as the basis for calculations (see Figure S2), with more than 20 Å of vacuum in the direction perpendicular to the surface. Note that the structure assumed for the model is the traditional one, displayed by most MXenes, in which the atomic layers of the MXene are stacked in an ABC fashion (Figure S3). Although the ABC stacking is metastable^40,45^ compared to the most stable ABA phase, it appears to match the Mo_2_C sample synthesized in the present case from Mo_2_Ga_2_C MAX etching. The ABC and ABA stacking phases can be easily distinguished by their lattice parameters, 2.99 Å and 2.84 Å, respectively,^41^ and the experimental value in our sample of 3.0 Å indicates that it corresponds to the selected model, in agreement with the layer stacking found in Mo_2_Ga_2_C. The selection of O surface terminations in our model is based on the XPS and EDX characterization.

A discrete grid with 3 × 3 × 1 special *k* points was used in Brillouin zone integrations, obtained according to the prescription of Monkhorst and Pack.^46^ The electron wave functions were treated as linear combinations of plane waves with an energy cutoff of 415 eV. The atomic positions were relaxed until the forces acting on all atoms were under 10 meV/Å, and the occupation of electronic orbitals was considered converged when the energy difference between two consecutive electronic iterations was lower than 10^–5^ eV. These parameters resulted from preliminary convergence tests.

The adsorption energies (*E*_ads_) were calculated as

1 $$E_{ads}=E_{surface+adsorbate}-E_{surface}-E_{adsorbate}$$

where *E*_surface_ and *E*_surface+adsorbate_ are the total energies of MXene models without and with an adsorbate, respectively, and *E*_adsorbate_ is the total energy of the adsorbate in a large asymmetric simulation box.

## Results and Discussion

### Synthesis and Characterization of Fe­(SA)/Mo_2_C

The parent MAX phase Mo_2_Ga_2_C was etched by in situ HF generation, which diminishes the safety hazards associated with handling concentrated HF.^47^ A mixture of HCl, NH_4_F and deionized water generates HF according to

2 $${NH}_{4}F+HCl\to {NH}_{4}Cl+HF$$

During the etching, Ga is removed and surface terminations install in a single step. The overall reactions can be expressed as

3 $${Mo}_{2}{Ga}_{2}C+6HF\to 2{GaF}_{3}+{Mo}_{2}C+3H_{2}↑$$

4 $${Mo}_{2}C+H_{2}O\to {Mo}_{2}CO+H_{2}↑$$

5 $${Mo}_{2}C+2HF\to {Mo}_{2}{CF}_{2}+H_{2}↑$$

As a result, the freshly produced MXene nanosheets bear predominantly −O and minor −F groups, which enhance hydrophilicity and provide colloidal stability to the MXene flake suspension in water. Iron single atoms, Fe­(SA), were subsequently incorporated by overnight impregnation of the etched MXene in an aqueous Fe^2+^ solution to yield Fe­(SA)/Mo_2_C. Inductively coupled plasma optical emission spectroscopy (ICP-OES) confirmed a nominal Fe loading of 0.6 wt %. Scheme 1 provides a pictorial illustration of the synthetic route used to obtain Fe­(SA)/Mo_2_C from its Ga-containing MAX precursor. No additional intermediate treatment specifically designed to create isolated islands or defects prior to Fe deposition was necessary. The HF-etched Mo_2_C MXene obtained from the MAX phase is known to contain intrinsic defects that can strongly interact with metal precursors, thereby enabling direct anchoring of Fe species on the MXene matrix.^48^ The distribution of Fe as isolated atoms or small clusters is further influenced by the concentration of the iron precursor in the impregnation solution, as demonstrated in our previous work.^49^

**Scheme 1:**
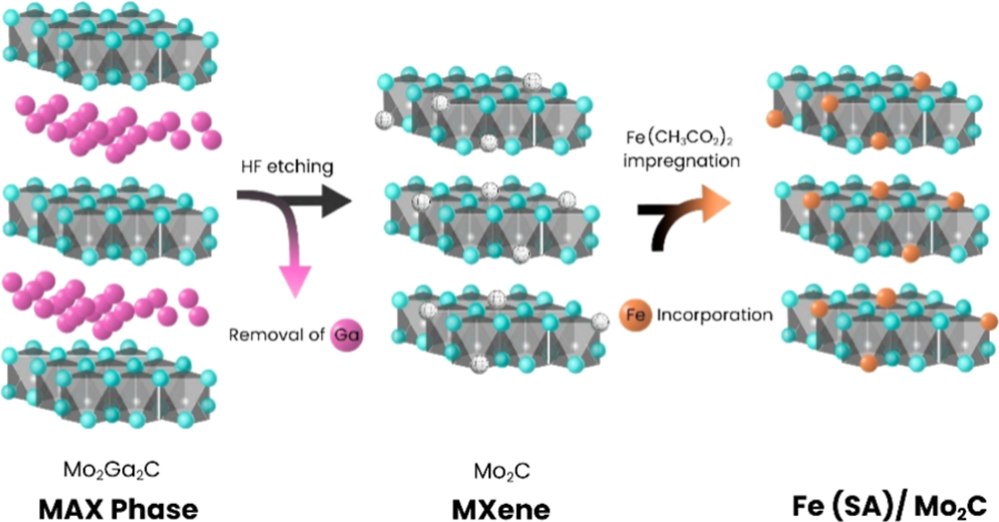
Schematic Illustration of the Synthesis of Fe Single Atoms (SA) Anchored on the Mo_2_C MXene Fe­(SA)/Mo_2_C. From Left to Right: (i) Selective Etching of Ga from the Mo_2_Ga_2_C MAX Phase via in Situ Generated HF (HCl/NH_4_F/H_2_O), Yielding Multilayer Mo_2_C MXene Sheets Terminated Predominantly with −O (and Minor −F) Groups; (ii) the Resulting MXene, Containing Intrinsic Vacancies, is Impregnated Overnight with an Aqueous Fe^2+^ Precursor, Enabling Direct Anchoring of Fe Species; (iii) formation of Fe­(SA)/Mo_2_C, where Fe is Stabilized as Isolated Atoms on the MXene Surface. Color Code: Mo (Green), Ga (Pink), Fe (Orange), Vacancies (white Spheres)

Powder X-ray diffraction (XRD) patterns of the parent MAX phase, the etched Mo_2_C MXene, and the Fe­(SA)-modified sample, Fe­(SA)/Mo_2_C, are presented in Figure 1. Upon etching, the (002) reflection shifts from 2θ ≈ 9.8° to 9.0°. This change is attributed to the removal of Ga and the formation of surface terminations. The intercalation of the etchant, together with the extraction of the A layer, induces an initial expansion of the MXene structure, which is further enhanced by the introduction of surface terminal groups. After Fe incorporation, the (002) peak shifts to lower angles and shows a slight decrease in intensity, indicative of partial delamination and increased structural disorder. This additional expansion is associated with the intercalation of the Fe ions between the layers during the impregnation process as well as the mechanical stress of stirring. No reflections attributable to Fe, FeO_*x*_, or other crystalline iron phases are detected, consistent with the low Fe loading and implying the absence of large Fe nanoparticles. A minor increase in background intensity, attributed to Fe-induced X-ray fluorescence under CuKα radiation, confirms the presence of Fe species within the material while preserving the overall α-Mo_2_C structure.

**Figure 1:**
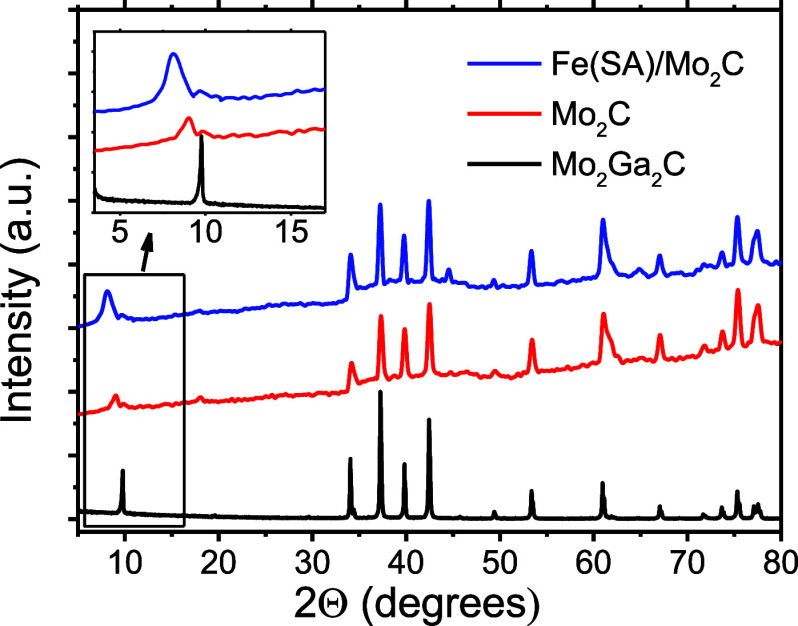
XRD patterns of the MAX phase (black), the etched MXene Mo_2_C (red) and Fe­(SA)/Mo_2_C MXenes (blue).

Atomic-force microscopy (AFM) height mapping (Figure S4) of drop-cast Fe­(SA)/Mo_2_C MXene flakes reveal a heterogeneous population of partially delaminated sheets with lateral sizes ranging from a few hundred nanometres to nearly 1 μm. Representative height profiles show the coexistence of both thin and thick domains: the red line in Figure S4 corresponds to a platelet of ∼25–40 nm thickness, while the pink, blue, and green traces indicate thinner regions between 3 and 20 nm. These values are characteristic of few-layer Mo_2_C nanosheets terminated with surface groups and slightly hydrated, together with residual multilayer stacks that have resisted exfoliation.

High-resolution transmission electron microscopy (HR-TEM) and aberration-corrected scanning transmission electron microscopy (AC-STEM) were employed to probe the atomic structure and Fe distribution in Fe­(SA)/Mo_2_C. The aberration-corrected annular dark-field (ADF) images (Figure 2a) display continuous lamellae extending over several tens of nanometers without signs of turbostratic disorder, confirming that the in situ HF etching yields well-defined Mo_2_C layers. The corresponding fast Fourier transform (FFT) pattern can be indexed exclusively to the α-Mo_2_C phase, demonstrating that the carbide lattice remains crystalline after etching. Clear gallery spacings with an average interlayer distance of 1.10 ± 0.1 nm are observed, in excellent agreement with the d(002) value from XRD. Each gallery appears as two parallel rows of bright atomic columns, which Z-contrast simulations assign to Mo atom rows viewed along the (0–1 0) zone axis. FFTs from the same region reproduce the simulated diffraction pattern of this projection, confirming the structural assignment. Additionally, lattice fringes with a periodicity of 0.26 nm are resolved, consistent with the characteristic interplanar spacing of Mo_2_C MXene. Side-view TEM images collected from multiple flakes (Figure S5) yield the same 1.1 nm spacing, verifying structural homogeneity across the specimen. The simultaneous observation of ordered Mo atomic columns and intact layer stacking provides compelling evidence of the structural integrity of the MXene after Fe incorporation. Characteristic hexagonal reflections in the FFT (highlighted in white circles in Figure 2b), further confirm preservation of the α-Mo_2_C framework.

**Figure 2:**
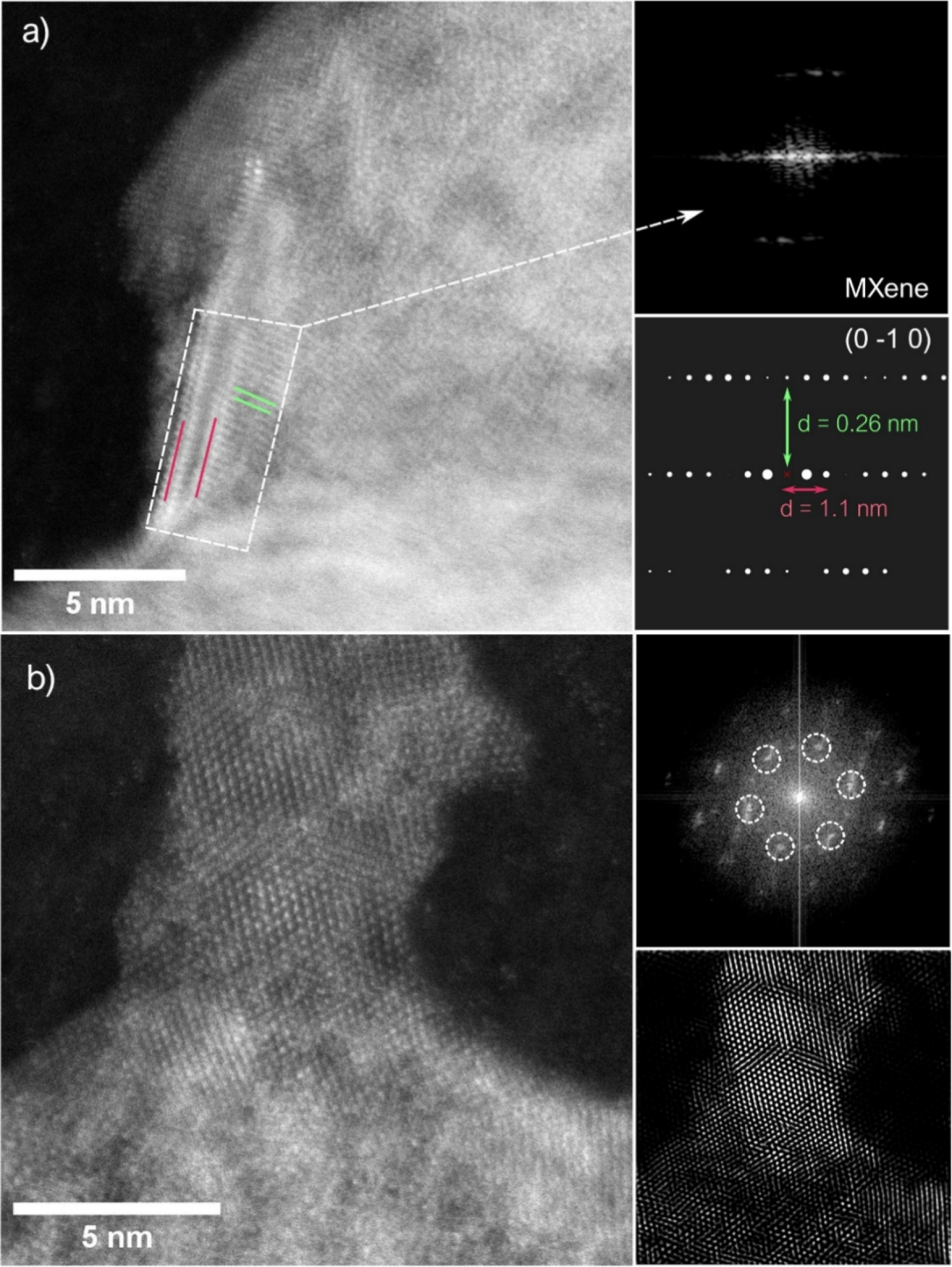
(a) HR-TEM image of Fe­(SA)/Mo_2_C and the respective Fourier transform (FFT) of the TEM micrograph inset. The lower panel represents the corresponding simulated diffraction pattern generated using the ReciPro software (b) HR-STEM image of Fe­(SA)/Mo_2_C. The FFT shows the typical hexagonal pattern, with *d* = 0.26 nm.

Additional ADF-STEM imaging of Fe­(SA)/Mo_2_C (Figure 3a) shows no evidence of Fe clustering or nanoparticle formation across the Mo_2_C terraces, indicating that Fe is not present as aggregated species. Elemental mapping (Figure 3b) further confirmed the uniform spatial distribution of Fe throughout the Mo_2_C matrix, with no Fe-rich aggregates, thereby providing strong support to the formation of atomically dispersed Fe single atoms, Fe­(SA), on the Mo_2_C MXene. In addition, element-specific STEM–EDX line profiling (Figure S6) revealed a homogeneous distribution of all constituent elements across the scanned flake region.

**Figure 3:**
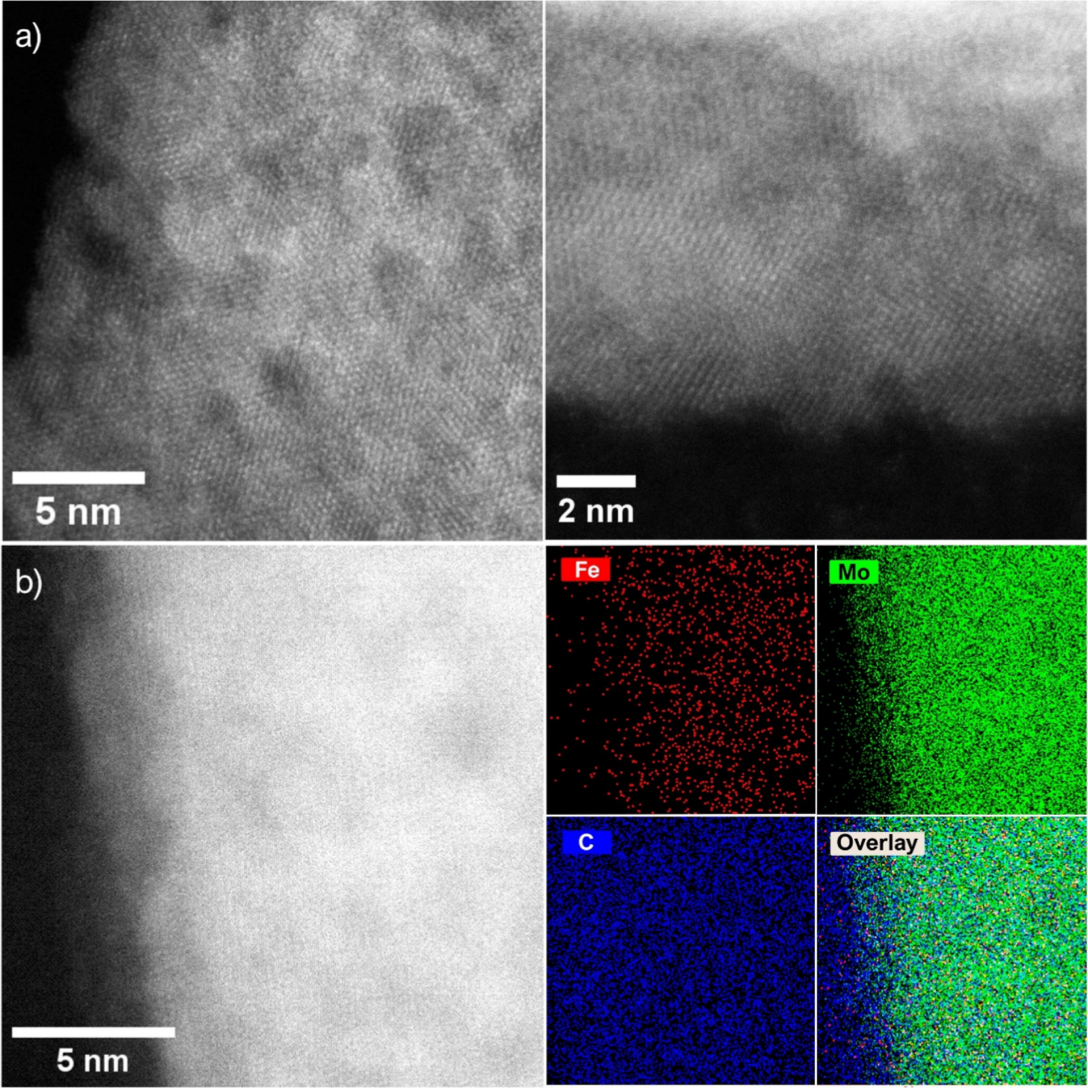
(a) ADF-STEM images of Fe­(SA)/Mo_2_C at two magnifications. (b) Elemental mapping of Fe­(SA)/Mo_2_C.

XPS analysis was performed to further examine the composition and chemical states of the elements in Fe­(SA)/Mo_2_C. As shown in Figure 4, the Mo 3d spectrum can be deconvoluted into two main doublets. The peaks at 229.6 eV (3d_5_/_2_) and 232.8 eV (3d_3_/_2_) are assigned to Mo–C species characteristic of Mo_2_C MXene.^50^ Notably, the binding energy of the Mo–C component at 229.6 eV is 1.5 eV higher than that of the Mo–C species at 228.1 eV reported for Mo_2_Ga_2_C,^51^ indicating that Ga atoms are replaced by electronegative surface terminations during the etching process.^52^ A second, weaker doublet at 232.7 eV (3d_5_/_2_) and 235.8 eV (3d_3_/_2_) corresponds to Mo^6+^ species.^53^ Although the Ga 2p XPS region was almost negligible (atomic proportion 0.05%), ICP-OES analysis confirms the presence of a low residual Ga content (Ga = 6.2 wt %), indicating that the etching process is largely effective, but it does not remove Ga completely. The O 1s region confirms that the surface of the Mo_2_C MXene is predominantly terminated by O-containing groups, with contributions assigned to C–O (533.4 eV), C–OH (532.4 eV), C=O (552.5 eV), and adsorbed water (533.6 eV). The peaks at lower binding energies are Mo–O and Fe–O, with peaks at 531 and 530.1 eV respectively. In contrast, only a weak signal is observed in F 1s region, indicating a low concentration of −F surface terminations (685.5 eV). This predominance of O-based functional groups is attributed to the extended HF etching conditions, which promote the substitution of fluorine terminations by oxygen-containing species through hydrolysis and surface restructuring.^54^ The C 1s spectrum shows four main contributions at 283.7, 284.5, 285.6, and 288 eV, corresponding to C–Mo, C–C, C–O/C–OH, and C = O species, respectively. Finally, the Fe 2p spectrum displays signals at ∼710.4 eV and ∼712.3 eV, characteristic of Fe^2+^ and Fe^3+^ species, respectively.

**Figure 4:**
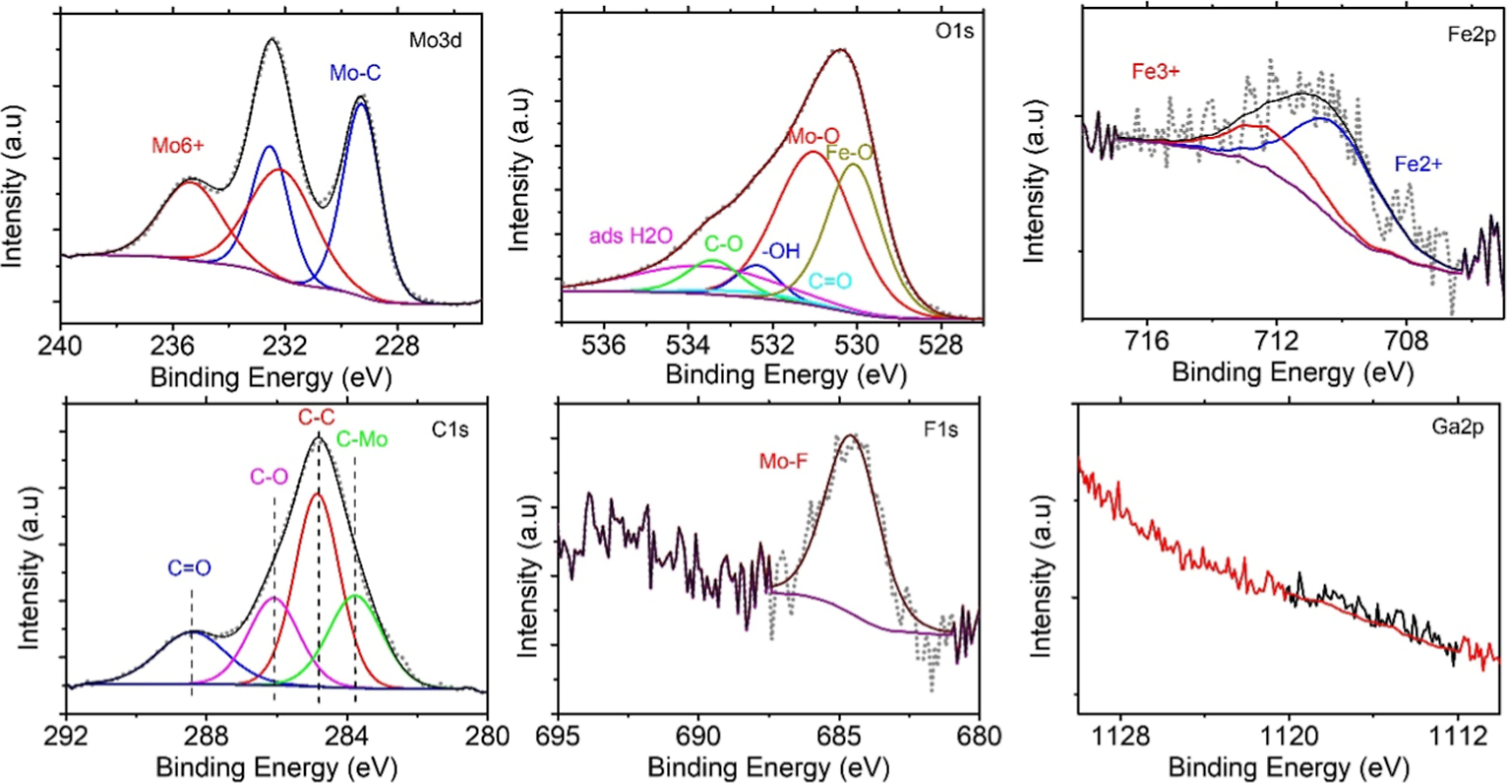
XPS analysis of Fe­(SA)/Mo_2_C.

To gain deeper insight into the chemical environment of the metal centers, particularly Fe, Fe K-edge XANES and EXAFS measurements were performed.^18,55–57^ The average oxidation state of iron in Fe­(SA)-Mo_2_C was determined from Fe K-edge X-ray absorption near-edge structure (XANES). Comparison of the Fe­(SA)-Mo_2_C spectrum with those of Fe, FeO, Fe_2_O_3_, and Fe_3_C references (Figure 5a) shows that the absorption edge position lies between FeO and Fe_2_O_3_, indicating an average Fe valence state between +2 and +3, in agreement with the XPS data.

**Figure 5:**
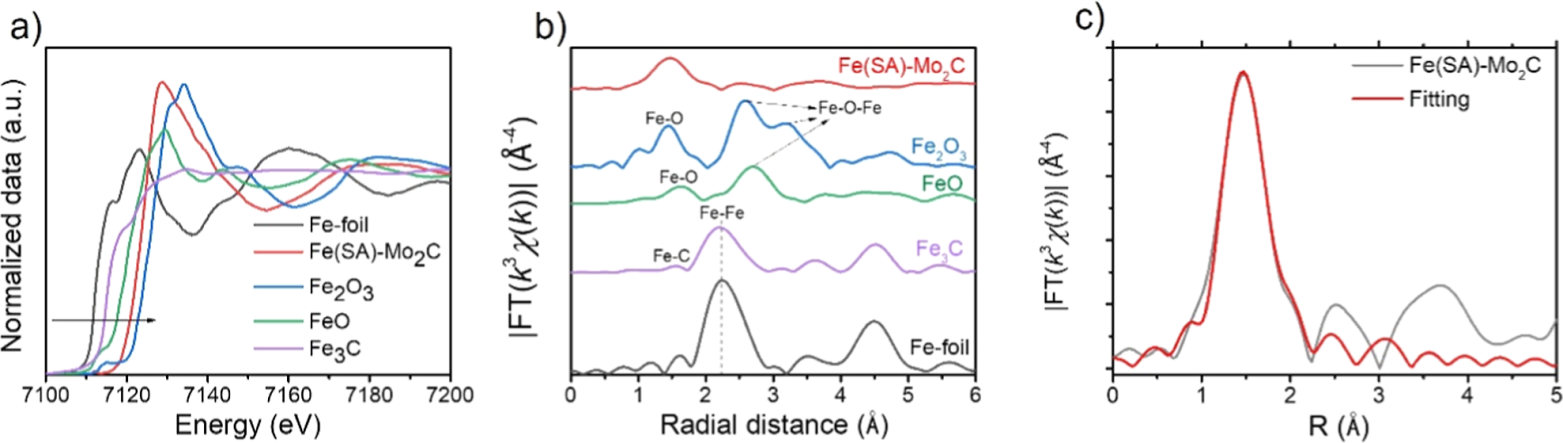
(a) XANES data at the Fe K-edge. (b) FT k^3^-weighted χ­(k)-function of the EXAFS spectra at the Fe K-edge. (c) Fitting results of the EXAFS spectra of Fe­(SA)-Mo_2_C in the R-space.

The local coordination environment of Fe was examined by Fourier-transform extended X-ray absorption fine structure (EXAFS) at the Fe K-edge (Figure 5b). In R-space, Fe­(SA)-Mo_2_C exhibits a prominent peak around 1.5 Å, corresponding to the first coordination shell of Fe–O/C. Notably, no characteristic Fe–Fe scattering peak (typically at ∼2.2 Å) was observed, ruling out the presence of Fe clusters and confirming the atomic dispersion of Fe species, consistent with the HAADF-STEM results. The corresponding Wavelet transform (WT) analysis (Figure S7) further identifies the backscattering atoms around Fe. The WT intensity maxima shift toward higher k values with increasing atomic number of the scatterer. In Figure S7, the observed maxima correspond to Fe–O/C coordination.

Quantitative EXAFS fitting (Figure 5c) using a structural model derived from DFT calculations (see below), where the Fe atom is filling a Mo vacancy (Figure S8, right), achieved good agreement with the experimental data (R-factor = 0.017). Additionally, alternative structural models in which the Fe atom is only adsorbed rather than substituting a Mo atom were also fitted. The corresponding models can be found in Figure S8, while the results of the fitting can be found in Figure S9 and Table S1 in the Supporting Information. As can be seen, only the model in which Fe substitutes a Mo atom offers a satisfactory fit. The fitting parameters (Table S1) reveal Fe–O, Fe–C, and Fe–Mo coordination environments, providing strong evidence that Fe atoms are incorporated into the Mo_2_C lattice by substitution at Mo sites. The good fitting of the EXAFS data validates the DFT-based structural model and confirmed the substitutional Fe-by-Mo configuration of Fe location in Fe­(SA)-Mo_2_C.

### Catalytic Reaction Evaluation

The catalytic performance of the as-prepared materials was first evaluated for the reduction of 4-NP to 4-AP in water at 60 °C, using an excess of NaBH_4_ as the reducing agent. The MXenes tested included Fe­(SA)/Mo_2_C and Mo_2_C as well as the Mo_2_Ga_2_C MAX phase for comparison. The reaction progress was monitored by UV–vis spectroscopy (Figure 6a), following the decay of the 4-nitrophenolate absorption band (λ = 400 nm) and the concomitant growth of the 4-AP band (λ = 300 nm). Observation of a clear isosbestic point in the series of UV–vis absorption plots indicate clean the transformation of 4-NP into 4-AP without the formation of biproducts. Kinetic analysis based on the Beer–Lambert law at 400 nm revealed that Fe­(SA)/Mo_2_C exhibits the highest catalytic activity in the series, reaching 4-NP nearly complete conversion of 4-NP with full selectivity toward 4-AP within 60 min with a pseudo-first-order rate constant of 0.0573 min^–1^ (Figure 6b) and turnover number (TON) and turnover frequency (TOF) values of about 45 and 5 min^–1^, respectively. This TOF value compares favorably with analogous MXene-based catalysts and even with other noble metal based solid catalysts (Figure S10). Furthermore, although a direct comparison of apparent activation energy (*E*_a_) values cannot be established because the studies corresponding to the catalysts included in Figure S10 do not report temperature-dependent kinetic analyses, the experimentally obtained *E*_a_ value of 120 kJ mol^–1^ for Fe­(SA)/Mo_2_C still offers valuable insight into the kinetics of the catalytic reduction process. Furthermore, at lower Fe­(SA)/Mo_2_C loadings, full 4-NP conversion was still achieved at extended reaction times, corresponding to a TON of 98. In contrast, Mo_2_C MXene and corresponding MAX phase show negligible activity, highlighting the important role of the supported iron species as the catalytically active sites (Figure 6b). Additionally, catalysts with different Fe single-atom loadings were evaluated alongside a control sample consisting of a physical mixture of iron oxides and Mo_2_C. As illustrated in Figure S11, a loading of 0.6 wt % was identified as the optimal iron content for the highest catalytic performance. The catalyst containing 0.3 wt % Fe showed lower activity, likely due to the lower density of active sites, whereas increasing the Fe loading to 1.0 wt % did not improve the catalytic behavior, possibly due to reduced site accessibility or the onset of incipient metal clustering. In comparison, a control sample consisting of a physical mixture of commercial Fe_3_O_4_ and Mo_2_C exhibited significantly lower catalytic activity than Fe­(SA, 0.6%)/Mo_2_C, indicating that the enhanced catalytic behavior cannot be attributed to the simple coexistence of iron oxide species and Mo_2_C. These observations support the key role of substitutional atomically dispersed Fe sites on the MXene surface in promoting the catalytic reaction. Another control experiment carried out in the absence of any catalyst revealed a negligible 4-NP conversion by NaBH_4_. PXRD of used solid catalysts showed that their initial crystallinity is retained upon use (Figure S12). ICP-OES analyses of the aqueous phase at the end of the reaction showed, in the case of Fe­(SA)/Mo_2_C, the absence of iron leaching, while some Mo (10 mg L^–1^, corresponding to 3.2% of the total Mo in the Fe­(SA)/Mo_2_C catalyst) was measured. To verify the heterogeneous nature of the reaction using Fe­(SA)/Mo_2_C as the catalyst, a hot-filtration test was performed (Figure 6b). Briefly, after the reaction was initiated (5 min), the solid catalyst was removed from an aliquot while the mixture was hot, and the filtrate was allowed to react further under identical conditions (60 °C). While the catalyst-free solution showed no additional conversion, which maintained the ∼45% conversion reached at 5 min, the remaining reaction mixture that still contained the solid catalyst proceeded to full conversion. These results indicate that the reaction proceeds exclusively via heterogeneous catalysis, with no significant contribution from leached homogeneous species.

**Figure 6:**
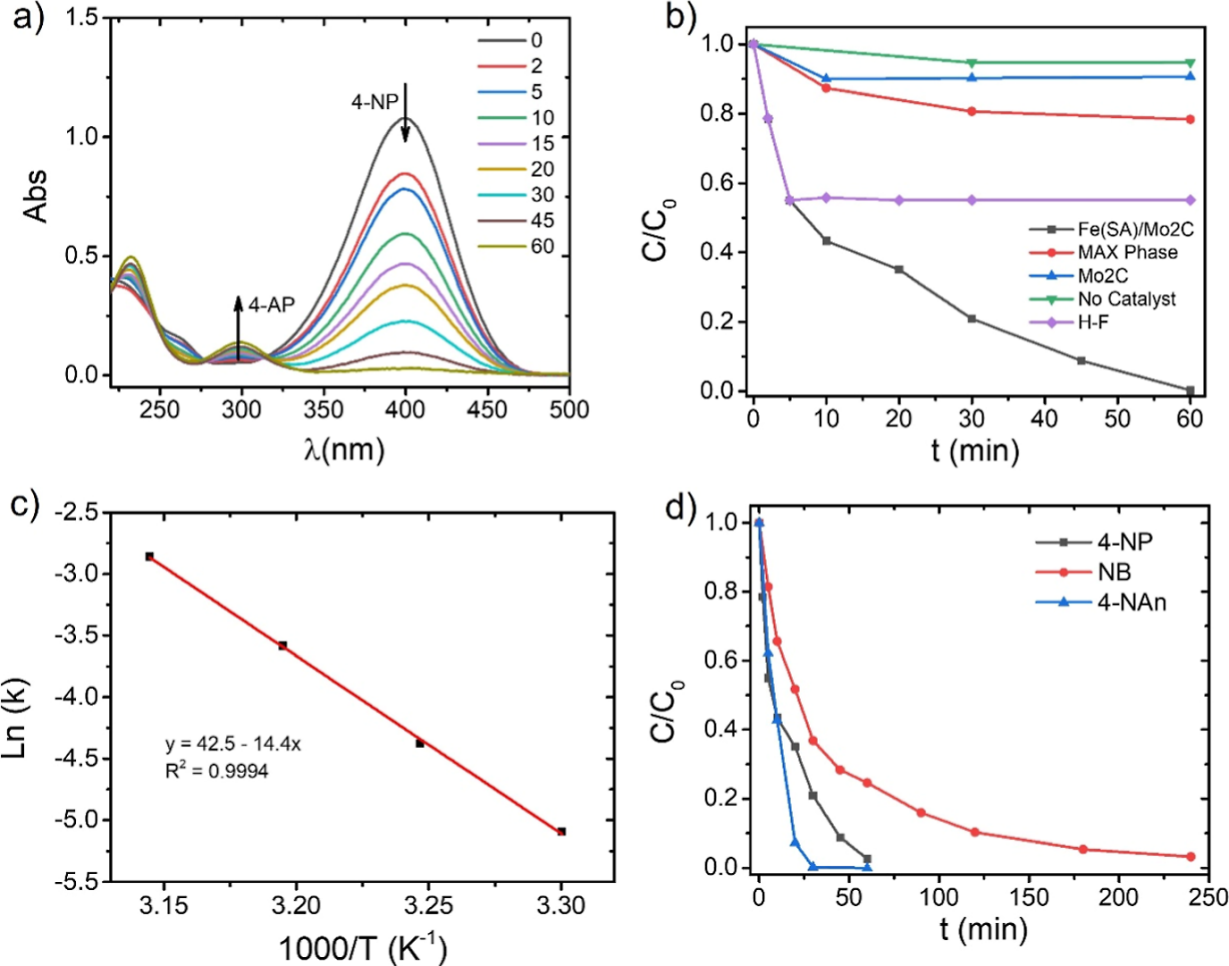
(a) UV–vis absorption spectra of the reaction solution at different reaction times using Fe­(SA)/Mo_2_C as catalyst for the reduction of 4-NP to 4-AP with NaBH_4_. (b) 4-NP conversion using Fe­(SA)/Mo_2_C, Mo_2_C and MAX phase as catalyst. A control experiment in absence of catalyst and the hot-filtration tests are also shown. (c) Arrhenius plot determined from 4-NP conversion as a function of the reaction temperature over Fe­(SA)/Mo_2_C. (d) Comparative catalytic activity of Fe­(SA)/Mo_2_C toward the reduction of three nitroarenes (4-nitrophenol = 4-NP, nitrobenzene = NB, and 4-nitroaniline = 4-NAn).

For the most active Fe­(SA)/Mo_2_C catalyst, the effect of reaction temperature (30–60 °C) on the kinetics was investigated, these data were also used for comparison with other catalysts in Figure S10. The results show an increase in reaction rate with temperature (Figure 6c). Arrhenius plot of the logarithm of the apparent rate constant gives an apparent activation energy (*E*_a_) of 120 kJ mol^–1^ (1.24 eV).

The catalytic versatility of Fe­(SA)/Mo_2_C was further tested for other two nitroarene derivatives as illustrated in Figure 6d. For nitrobenzene, which lacks the electron-donating group on the ring, the reaction rate decreased (0.028 min^–1^) respect to 4-NP (0.60 min^–1^). Conversely, 4-nitroaniline, featuring a highly electron-donating amine group, exhibited an accelerated rate with a *k* of 0.085 min^–1^.^58^

The catalytic activity and stability of Fe­(SA)/Mo_2_C was assessed over five consecutive cycles. Although full conversion was maintained across the initial runs, a noticeable decline in activity was observed by the fifth cycle, with the catalyst retaining 95% of its initial activity (Figure 7a). ICP analysis of the aqueous phase revealed negligible iron leaching; however, 30% cumulative molybdenum leaching was detected, which is likely to contribute to the observed deactivation. Furthermore, XRD analysis of the recovered catalyst (Figure 7b) showed new diffraction peaks and intensity shifts compared to the fresh sample, suggesting structural transformations. The (002) reflection shifts from 2θ ≈ 8.9° in the fresh Fe­(SA)/Mo_2_C to 7.2° after catalysis, corresponding to an increase in interlayer spacing from 0.99 to 1.23 nm during the reaction. A control experiment performed by mixing fresh Fe­(SA)/Mo_2_C with NaBH_4_ under identical reaction conditions, but in the absence of 4-NP, exhibited the same XRD peak shift (Figure S13), confirming that the observed expansion of the MXene interlayer distance is not related to nitroarene reduction but to the effect of NaBH_4_ and the reaction environment. Finally, TEM imaging of the five times used Fe­(SA)/Mo_2_C catalyst revealed the presence of some amorphous regions together with other regions with the typical hexagonally shaped FFT of MXene (Figure S14), meaning that partial crystallinity degradation is occurring under the reaction conditions.

**Figure 7:**
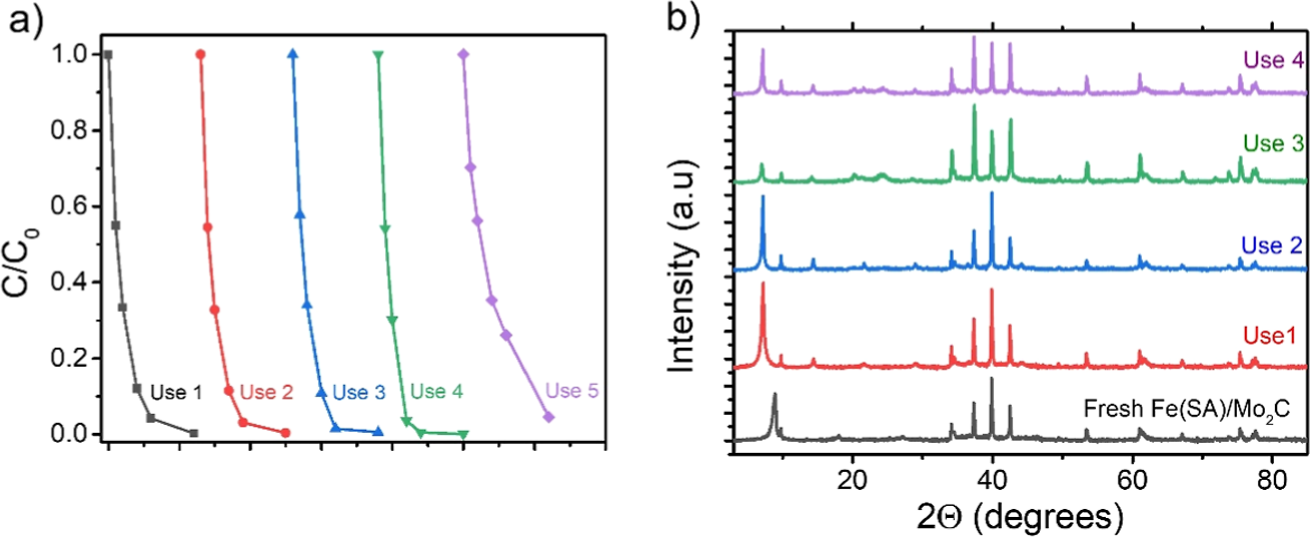
(a) Reusability tests of Fe­(SA)/Mo_2_C as catalyst for NP reduction. (b) X-ray diffractograms of Fe­(SA)/Mo_2_C before and after catalytic tests.

XPS analysis of the five times used Fe­(SA)/Mo_2_C shows that while the C 1s and Mo 3d peaks were similar, the O 1s shows an increase in the proportion of the –OH and Fe–O/Mo–O contributions (Figure S15). Interestingly, quantitative analysis of the elemental composition indicates an increase in the percentage of –OH from 1.5 to 10.4% and C–O from 11 to 17% from the fresh sample to the exhaustively used Fe­(SA)/Mo_2_C catalyst. These changes accompanied by a decrease in the Mo 3d proportion from 12.79 to 2.3% are compatible with the deposition of polymeric phenolic species on the Fe­(SA)/Mo_2_C surface upon its extensive use as catalyst. Also noticeable is the emergence of weak Ga signal in the used Fe­(SA)/Mo_2_C sample from the initial barely detectable 0.05% to 0.24% in atomic proportion. This indicates the exposure of some initially buried Ga species upon use of Fe­(SA)/Mo_2_C as catalyst, probably due to structural rearrangement and amorphization under the reaction conditions.

### DFT Calculations and Reaction Mechanism

In order to build a model of the catalyst that is as realistic as possible, we began by analyzing the most stable position of an Fe atom on the surface of the Mo_2_CO_2_ MXene (Figure S2). According to our XRD, HAADF-STEM and EXAFS analyses, Fe is present as dispersed single atoms, and the Fe doping does not change the overall structure of the MXene. Thus, we compared the energy of three models, each containing one Fe atom in a different position on the surface of the Mo_2_C MXene, as seen in Figure S8, and found that the most stable position is at a Mo vacancy. Notice that the substitutional Fe atom is barely visible in the side view, but a slight dislocation of the surrounding O atoms can be seen, accompanied by very small surface relaxation energy, −0.27 eV. This is in line with the observed conservation of the MXene structure upon doping with Fe. The Fe–Mo coordination distance obtained from EXAFS analysis (2.95 Å, Table S1) is similar to the values predicted by DFT. In the single-Fe model, an average Fe–Mo distance of 2.84 Å to the neighboring Mo atoms was obtained, while in the two-Fe model, distances in the range 2.81–2.85 Å were found. Furthermore, the XRD patterns of Fe-containing and pristine samples are essentially identical, indicating that the long-range MXene structure is preserved upon Fe incorporation. Taken together, these results support a structural model in which Fe atoms substitute Mo sites within the MXene lattice.

We measured the interaction between substitutional Fe atoms (Fe_Mo_) on the Mo_2_CO_2_ MXene from the thermodynamic point of view, by comparing the energy of two nearest-neighbor Fe_Mo_ with that of two Fe_Mo_ in different supercells. Here, the calculations showed that two Fe_Mo_ defects repel each other slightly, since bringing them together leads to a potential energy increase of 0.39 eV. Since this is a sizable amount of energy, but not thermodynamically prohibitive, we built and analyzed two separate catalyst models: one with a single Fe_Mo_ (the Fe@Mo_2_CO_2_ model), and another with two neighboring Fe_Mo_ (the Fe_2_@Mo_2_CO_2_ model).

It is known that the Mo_2_C MXene with partially removed surface termination is an active and selective catalyst for hydrogenations, as has been observed experimentally.^35,37^ Using DFT it has been predicted that the hydrogenation activity would be even enhanced if the MXene displays ABC stacking.^40^ In fact, the partial functionalization of MXenes in general is crucial to control their reactivity.^59,60^ Therefore, we compared the energetic cost to remove an O atom from the surface termination on a pristine Mo_2_CO_2_ model with that on the Fe@Mo_2_CO_2_ model. According to our calculations, having a substitutional Fe atom facilitates the removal of the neighboring surface termination of the MXene, by decreasing the required energy by 0.72 eV per O atom. This is very promising for catalysis as Fe doping (i) promotes the formation of small termination-free islands, which have been linked to the most active sites on the Mo_2_C MXene,^40,61^ and (ii) makes it easier to desorb O-containing species that may result from the reduction of 4-NP. The requirement of surface termination removal for molecule activation is illustrated in Figure S16. This Figure shows the adsorption configuration of 4-NP on a region of the Mo_2_CO_2_ model with a substitutional Fe atom, in the presence or absence of the three atoms of the surface termination that surround the Fe atom. The molecule can only interact directly with the surface metal atoms and become activated if the surface termination is partially removed. From here onward, our models assume the absence of O surface termination in the immediate vicinity of the substitutional Fe atoms.

We investigated the mechanisms and energetics of both BH_4_^–^ (modeled as a neutral BH_4_ species because of the limitations in handling charged systems with periodic DFT) dissociation and 4-NP reduction to 4-AP, on the Fe@Mo_2_CO_2_ model and on Fe_2_@Mo_2_CO_2_ model since these two reactions are expected to occur side by side on the surface of the MXene.

Concerning BH_4_ dissociation, the energies of all the adsorbed reaction intermediates were calculated using the infinite distance approach, following the methodology described in previous works,^62,63^ to keep the total number of atoms of each chemical element constant throughout the successive dehydrogenations. The potential energy diagram is shown in Figure 8 for both the Fe@Mo_2_CO_2_ (Fe­(SA)@Mo_2_C) and the Fe_2_@Mo_2_CO_2_ (Fe­(DA)@Mo_2_C) models. On both models, the first three dehydrogenations are exothermic or moderately endothermic. Clearly, the rate-determining step is the last one, BH* → B* + H* (where the * symbol stands for an adsorbed species), which is endothermic by around 0.6 eV on either model, although the effect is still considerably smaller than that observed on metal surfaces.^62^

**Figure 8:**
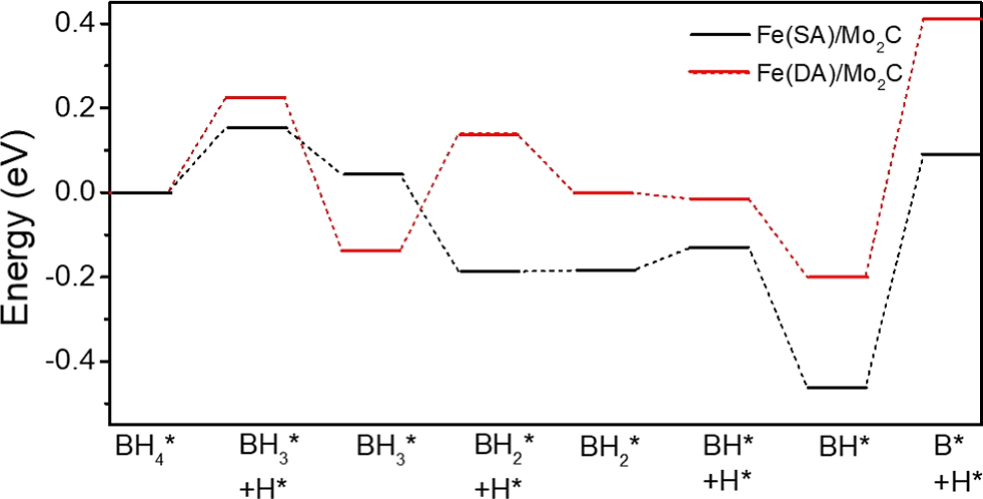
Potential energy landscape of BH_4_ dissociation on the Fe@Mo_2_CO_2_ (Fe­(SA)@Mo_2_C, black) and Fe_2_@Mo_2_CO_2_ (Fe­(DA)@Mo_2_C, red) models of the Fe-doped Mo_2_CO_2_ MXene surface.

To assess the broader relevance of the catalytic behavior beyond NaBH_4_-driven conditions, the interaction of H_2_ with the modeled surfaces was also investigated. The results indicate spontaneous dissociative adsorption of H_2_, with no detectable activation barrier, leading to the formation of adsorbed hydrogen atoms. This suggests that the same active sites can operate under H_2_-driven hydrogenation conditions, thereby extending the applicability of the catalyst beyond diffusion-controlled model reactions.

Our study of 4-NP reduction on the Fe@Mo_2_CO_2_ and Fe_2_@Mo_2_CO_2_ models began by assuming a dissociative mechanism, wherein 4-NP dissociates twice, resulting in a HO–C_6_H_4_–N species, which is then hydrogenated twice, ultimately yielding the desired 4-AP species. The corresponding energy diagram is presented in Figure 9c. The plots of the two models are nearly identical, both qualitatively and quantitatively. The two dissociative steps, HO–C_6_H_4_–NO_2_ → HO–C_6_H_4_–NO + O and HO–C_6_H_4_–NO → HO–C_6_H_4_–N + O, are exothermic by more than 2.5 eV, as expected, since MXenes excel at dissociating all kinds of chemical species, especially when O atoms are involved. However, the first hydrogenation, HO–C_6_H_4_–N + H → HO–C_6_H_4_–NH-, is quite energy-demanding, by 0.91 eV on Fe@Mo_2_CO_2_ or 1.21 eV on Fe_2_@Mo_2_CO_2_, hinting that a dissociative mechanism of 4-NP reduction may not be favored in this material. This result prompted us to consider another mechanism.

**Figure 9:**
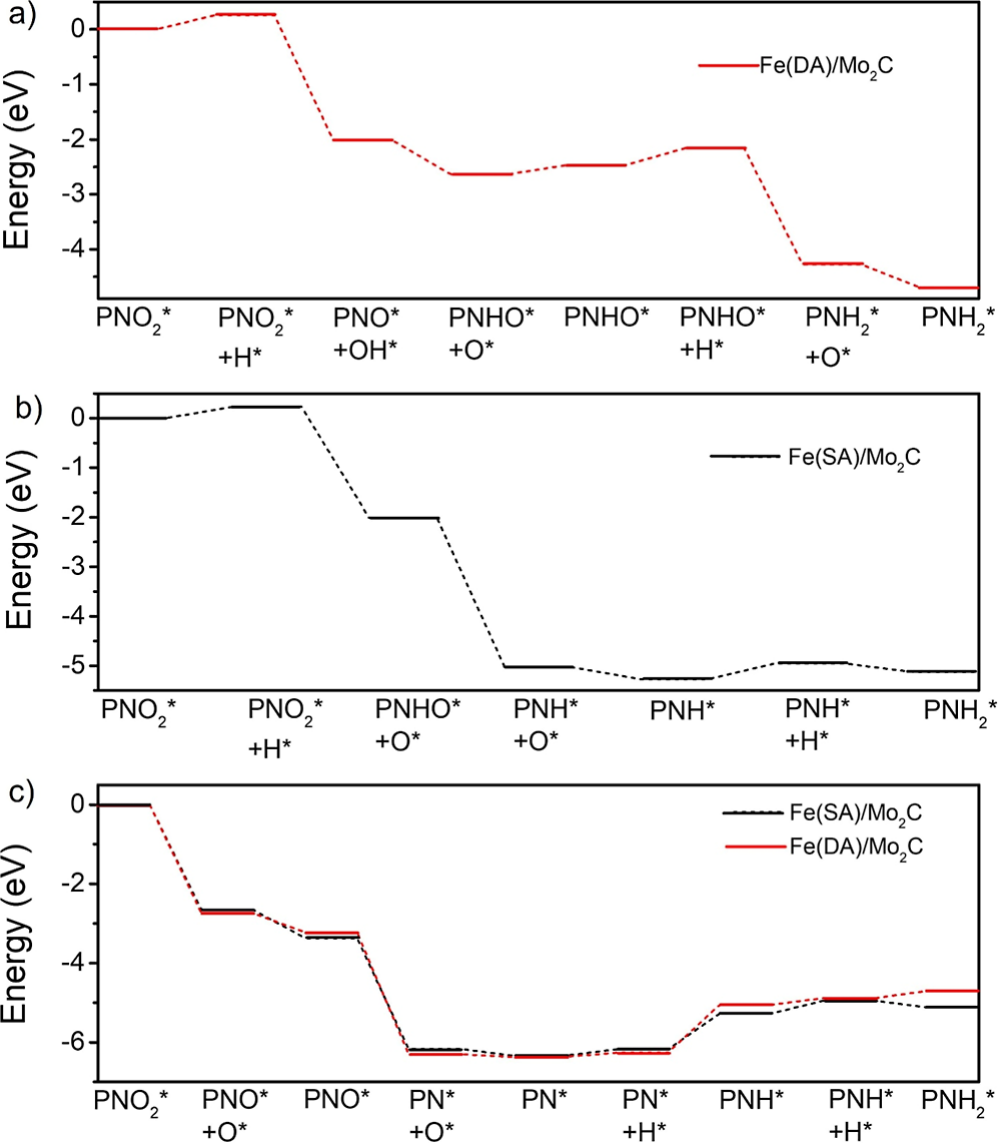
Potential energy landscape of 4-NP reduction to 4-AP on the Fe@Mo_2_CO_2_ (Fe­(SA)@Mo_2_C, black) and Fe_2_@Mo_2_CO_2_ (Fe­(DA)@Mo_2_C, red) models of the Fe-doped Mo_2_CO_2_ MXene surface, following (a,b) an associative mechanism., and (c) a dissociative mechanism.

In a previous study,^64^ an associative mechanism for the reduction of nitroaromatics is presented, in which the reaction proceeds by transferring H atoms from the catalyst surface to the O atoms of the nitro group, forming intermediates such as R–NOOH and weakening the N–O bonds. We investigated the possibility of 4-NP reduction following such an associative mechanism on the Fe@Mo_2_CO_2_ and Fe_2_@Mo_2_CO_2_ models. The respective energy diagrams are given in Figure 9a,b. The reaction steps for Fe@Mo_2_CO_2_ and Fe_2_@Mo_2_CO_2_ are different and are analyzed in detail in the following paragraphs.

Normally, the transfer of an H atom from the surface to the nitro group of 4-NP would form an ArNOOH intermediate. However, on the Fe@Mo_2_CO_2_ model, this transfer weakens the N–O bond sufficiently that it spontaneously breaks, separating the molecule into HO–C_6_H_4_–NO + OH. Furthermore, the adsorbed OH group is also unstable and spontaneously donates its H atom toward HO–C_6_H_4_–NO, forming HO–C_6_H_4_–N­(H)O + O. This process is illustrated in Figure 10a. The HO–C_6_H_4_–N­(H)O + O complex is stabilized by an NH···O hydrogen bond, with a length of 1.7 Å and an N–H–O angle of 152°. Once the O adatom leaves the vicinity of HO–C_6_H_4_–N­(H)O (in the form of water, for example), the HO–C_6_H_4_–N­(H)O species is no longer stable and dissociates into HO–C_6_H_4_–NH + O. The remaining step of the mechanism is the second hydrogenation, HO–C_6_H_4_–NH + H → 4-AP.

**Figure 10:**
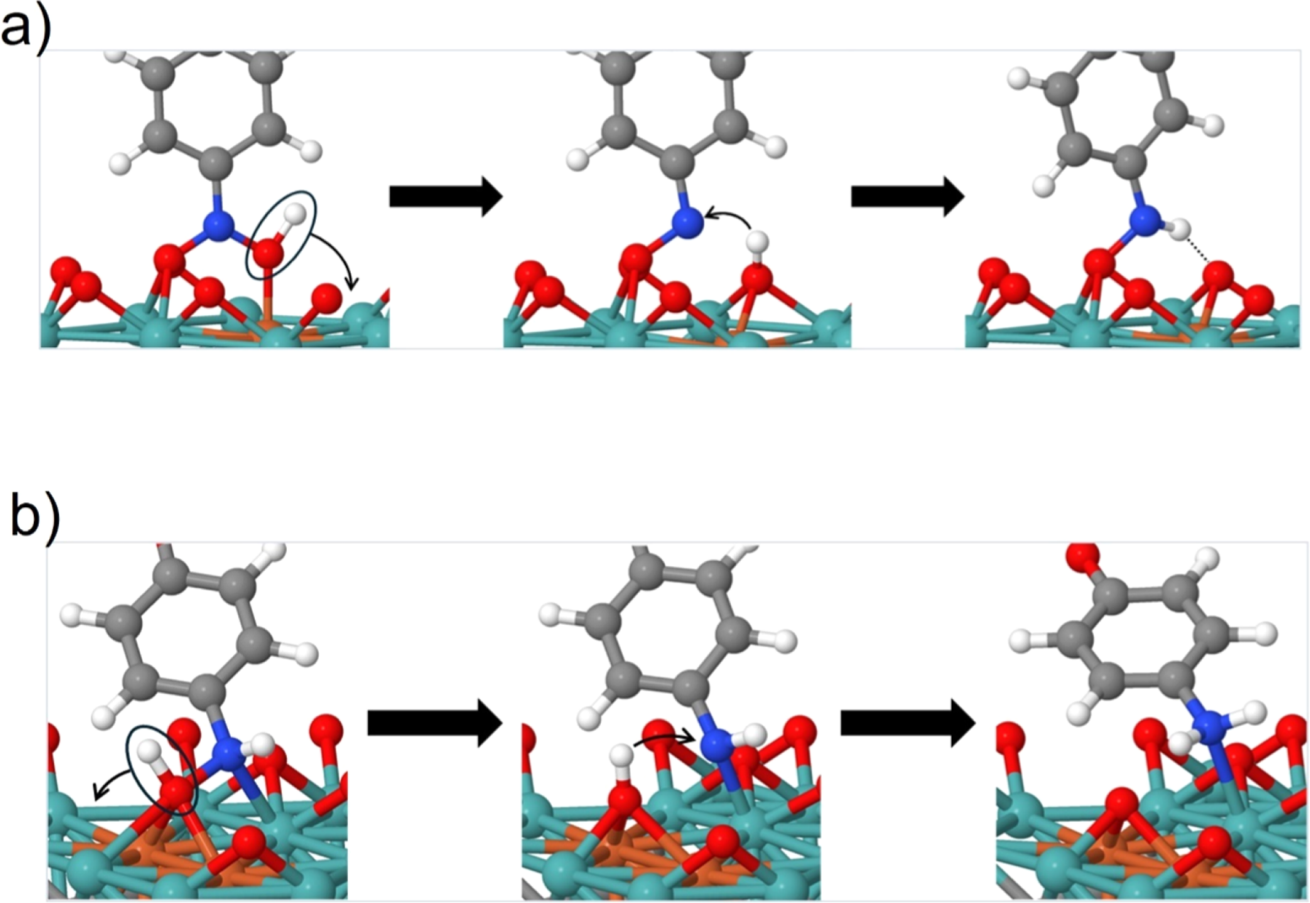
Illustration of the instability of the HO–C_6_H_4_–NO­(OH) species when adsorbed on (a) Fe@Mo_2_CO_2_ and (b) Fe_2_@Mo_2_CO_2_ (panels on the left), which spontaneously dissociate into HO–C_6_H_4_–NO and OH (central panels), and the latter donates their H atom, resulting in HO–C_6_H_4_–N­(H)O and O (right panels) Color code: Mo atoms in blue, C in gray, O in red, Fe in orange, N in blue, H in white.

On the Fe_2_@Mo_2_CO_2_ model, we found a distinct 4-NP reduction mechanism compared to the one on Fe@Mo_2_CO_2_. The first hydrogenation of the nitro group, HO–C_6_H_4_–NO_2_+H, spontaneously breaks one N–O bond, forming HO–C_6_H_4_–NO + OH. The subsequent H transfer is exothermic by −0.63 eV and forms HO–C_6_H_4_–N­(H)O + O. In the case of the second hydrogenation, the intermediate HO–C_6_H_4_–N­(H)­OH species is not formed. Instead, this species spontaneously dissociates into HO–C_6_H_4_–NH + OH, and the OH also spontaneously transfers its H atom to PNH, resulting in PNH_2_. This process is shown schematically in Figure 10b.

To assess the effect of temperature, we recalculated the energy landscapes considering Gibbs free energy (Δ*G*) instead of energy (Δ*E*), by correcting the energy of each reaction step as^65^

ΔG=ΔE+ΔZPE−TΔS

where ΔZPE is the change in zero-point energy, *T* = 333 K (60 °C is the temperature considered in the catalytic reaction evaluation, see above) is the absolute temperature, and Δ*S* is the change in entropy. We found that the change in the Gibbs free energy for each reaction step differs from its respective change in energy by at most 0.06 eV, across all reactions and surface models. In the case of 4-NP reduction (Figure 9), this corresponds to very negligible changes, since the energies involved are of the order of up to 6 eV. In the case of BH_4_ dissociation (Figure 8), the effect is slightly more noticeable, but still very small. In Figure S17, we show a comparison between the Δ*E* and Δ*G* plots in the case of the Fe@Mo_2_CO_2_ model. Since the temperature used in the calculations matches the highest temperature used in the catalysis tests, then the calculated differences between energy and Gibbs free energy were maximized. Therefore, if the calculations had considered temperatures lower than 333 K, these differences would be even smaller.

The results presented in the previous paragraphs tentatively explain the advantage of Mo_2_C as support of substitutional Fe single atoms and their superior catalytic activity. When compared to the dissociative mechanism, the associative mechanism allows the N–O bonds to still be broken easily, while hydrogenation steps become exothermic, due to the instability of OH groups on the surface of MXenes. In both single-Fe and double-Fe models, the spontaneous dissociations and hydrogen transfers contribute to a highly favorable associative pathway (Figure 9a,b). To further support this DFT-calculated pathway, ^1^H NMR spectroscopy studies monitoring the reaction progress were conducted (Figure S18a). The spectra reveal the progressive conversion of 4-NP into 4-AP, while no detectable signals attributable to reaction intermediates were observed during the measurements (Figure S18b,c). Although these experiments do not allow direct identification of highly transient or surface-bound intermediates, the absence of accumulated intermediate species is consistent with their rapid evolution toward the final product, in agreement with the fast kinetics and low activation barriers predicted by the DFT calculations.

## Conclusions

The present study demonstrates the potential of MXenes, particularly Mo_2_C, as supports of single-atom catalysts through substitutional replacement of Mo lattice atoms by Fe. The lattice location of Fe single atoms is experimentally supported by EXAFS. While the parent pristine Mo_2_C is catalytically inert for reduction of nitro aromatics by NaBH_4_, Fe­(SA)/Mo_2_C exhibits an excellent catalytic activity. Based on the pseudo-first order rate constant (*k* = 0.06 min^–1^), Fe­(SA)/Mo_2_C ranks among the best solid catalysts reported to date. Although Fe single atoms remain atomically dispersed and resistant to leaching under reaction conditions, the Mo_2_C MXene support undergoes partial structural degradation and Mo leaching during consecutive catalytic cycles. Future work will therefore focus on improving MXene structural robustness under reducing environments, for instance through surface termination optimization to enhance MXene robustness, structural stabilization strategies to limit Mo dissolution, or adjustment of reaction conditions to mitigate reductive degradation.

## Supplementary Material



## Notes

STEM and HR-STEM images; AFM measurements; STEM–EDX line scan analysis; Wavelet Transform plot of the Fe K-edge; comparative TOF values for the reduction of 4-NP to 4-AP; XRD, XPS, and TEM images after reuses; views of the MXene model, the three locations considered for an Fe atom, and the adsorption configurations of 4-NP (PDF)
