# Supplementary material for: Substitutional Single Iron Atoms as Active Sites for Nitrophenol Reduction by the Mo2C MXene
Source: ACS Appl Nano Mater. 2026 Jul 9;9(28):13589–602. doi: 10.1021/acsanm.6c02370 (PMC13386466; doi:10.1021/acsanm.6c02370)
Supplement: Supplementary file 1 [file an6c02370_si_001.pdf]

Supporting Information

Substitutional Single Iron Atoms as Active Sites for Nitrophenol Reduction by the Mo_2C MXene

Sabine Eliane Midré,[a] Victor Ramón-Trimiño,[b] Antón López-Francés,[b] Sergio Navalón,[b] José D. Gouveia,[c] José R. B. Gomes,[d] Sara Goberna-Ferrón,[a,*] Hermenegildo García,[a,*] Ana Primo[a,*]

[a] Instituto Universitario de Tecnología Química, Consejo Superior de Investigaciones Científicas-Universitat Politècnica de València, Universitat Politècnica de València, Av. De los Naranjos s/n, 46022 Valencia, Spain.

[b] Departamento de Química, Universitat Politècnica de València, Av. De los Naranjos s/n, 46022 Valencia, Spain.

[c] CICECO – Aveiro Institute of Materials, Department of Physics, University of Aveiro, Campus Universitário de Santiago, 3810-193 Aveiro, Portugal

[d] CICECO – Aveiro Institute of Materials, Department of Chemistry, University of Aveiro, Campus Universitário de Santiago, 3810-193 Aveiro, Portugal

Sara Goberna-Ferrón[a,*], Email: sgobfer@itq.upv.es

Hermenegildo García [a,*], Email: hgarcia@itq.upv.es

Ana Primo [a,*], Email: aprimoar@itq.upv.es

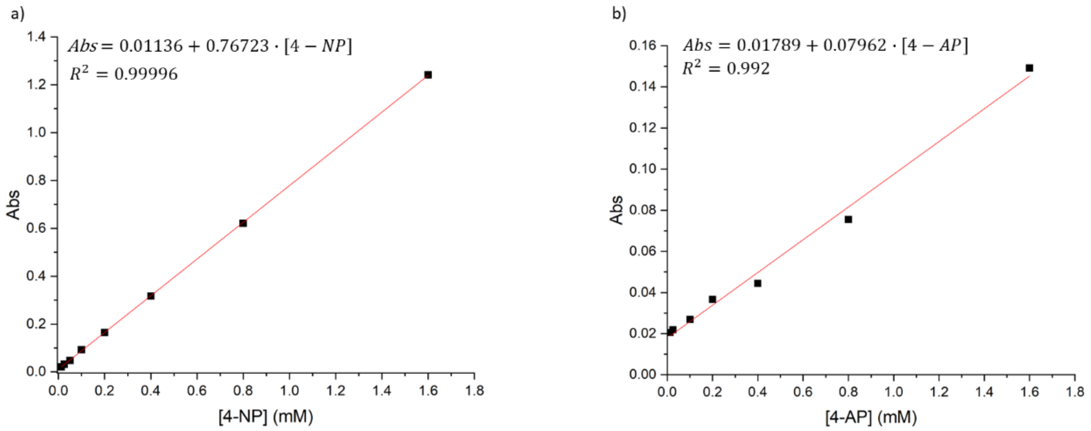

Figure S1. *UV–vis calibration curves of a) 4-nitrophenol (4-NP) and b) 4-aminophenol (4-AP) employed for quantification of substrate conversion and product formation during the catalytic reaction.*

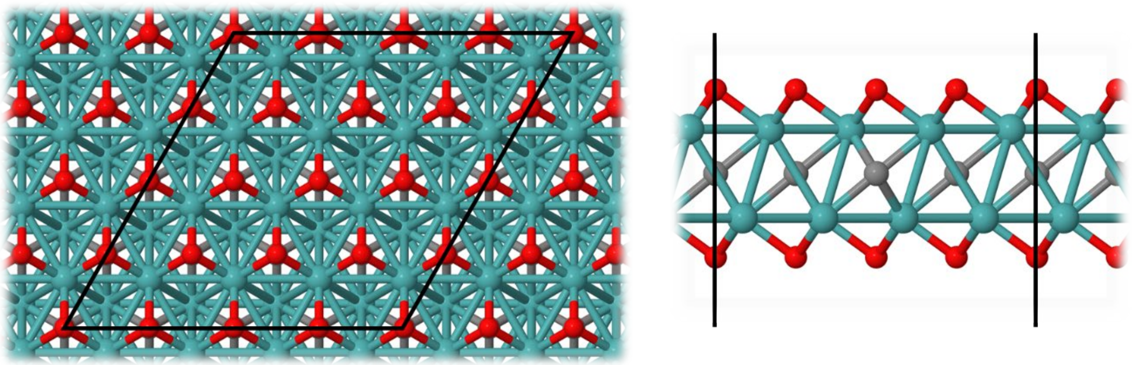

Figure S2*. Frontal (left panel) and side (right panel) views of the Mo_2CO_2 MXene model used in this work. The black lines represent the boundaries of the simulation supercell. Color code: Mo atoms in blue, C in grey, O in red.*

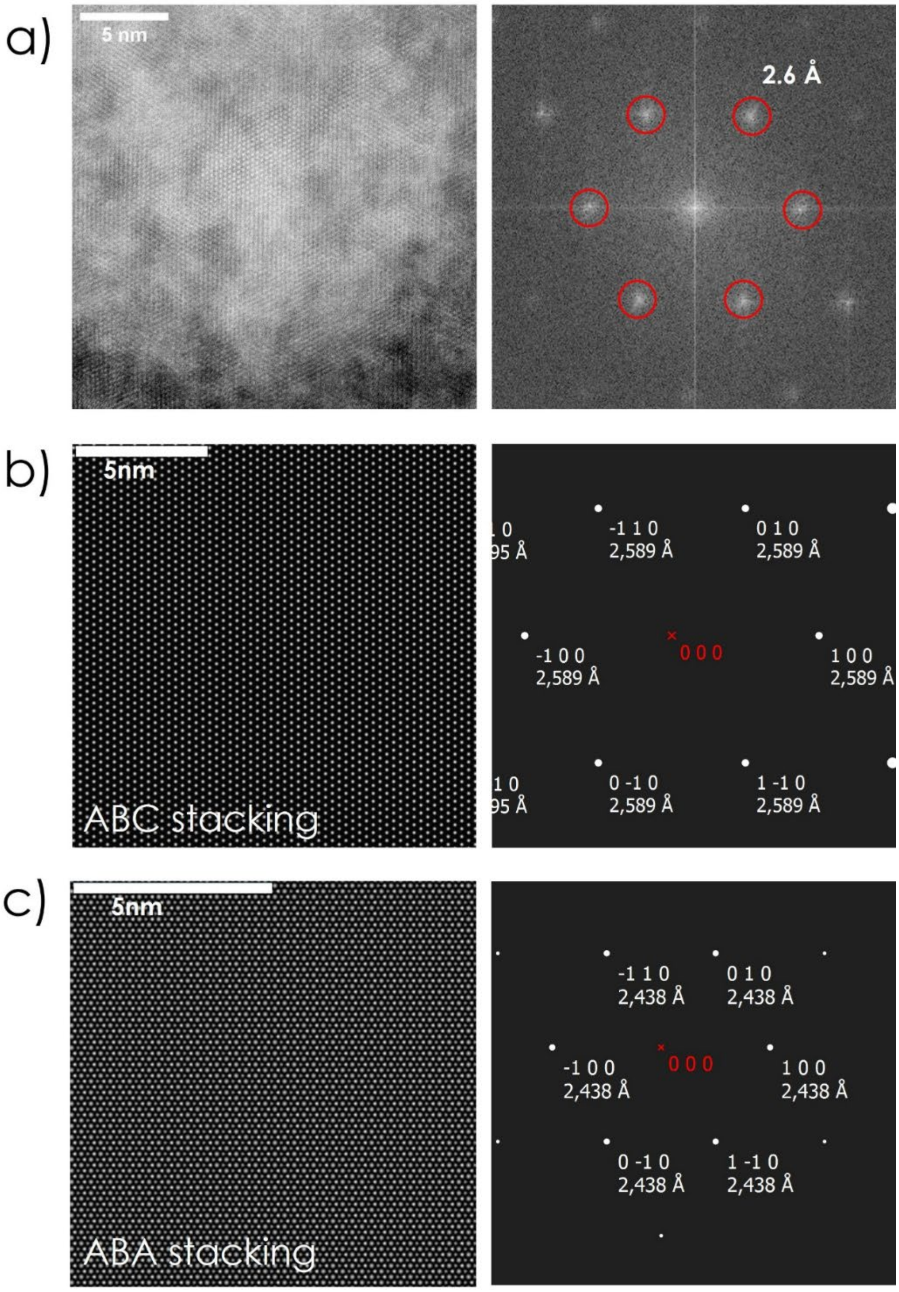

Figure S3. *Comparison of STEM images of the as synthesized Fe(SA)/Mo_2C and its respective FFT with the simulated Mxene in ABC and ABA stacking in the same orientation.*

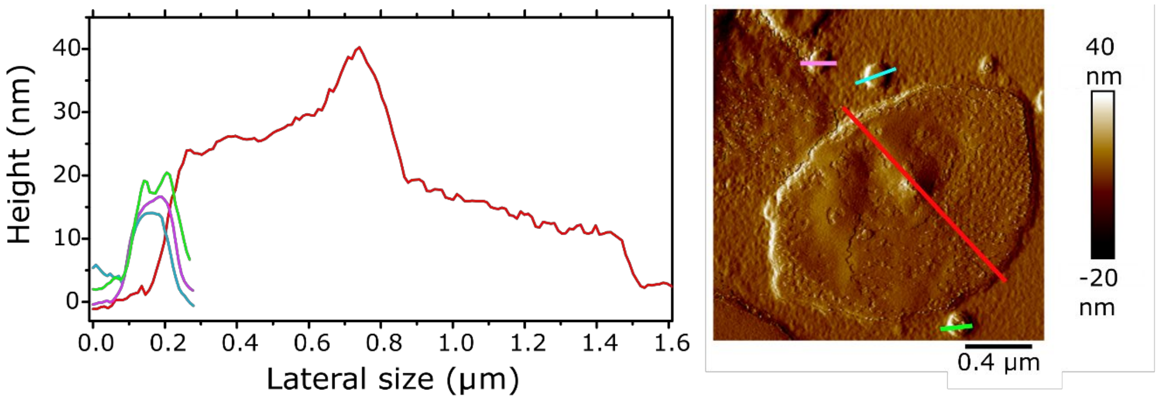

Figure S4. *AFM measurements of particle thickness for Fe(SA)/Mo_2C showing partial delamination and different sized flakes.*

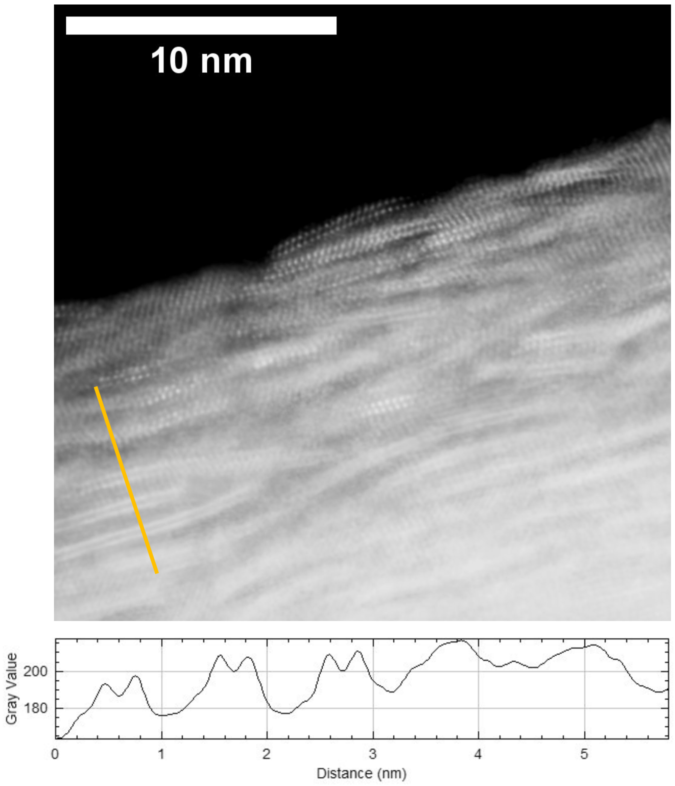

Figure S5. *HR-STEM image of Fe(SA)/Mo_2C, showing a side view of the layers, with the profile plotted below. The interlayer distance is shown to be 1.1 nm.*

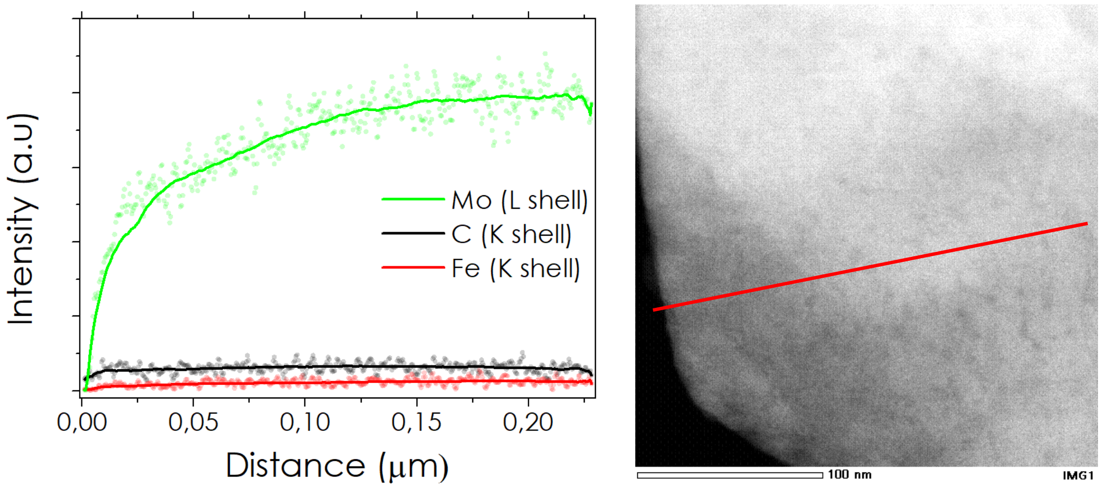

Figure S6. *STEM–EDX line scan across a Fe(SA)/Mo_2C flake (right, ADF -STEM image). Elemental profiles (left) show C (black), Mo (green), and Fe (red).*

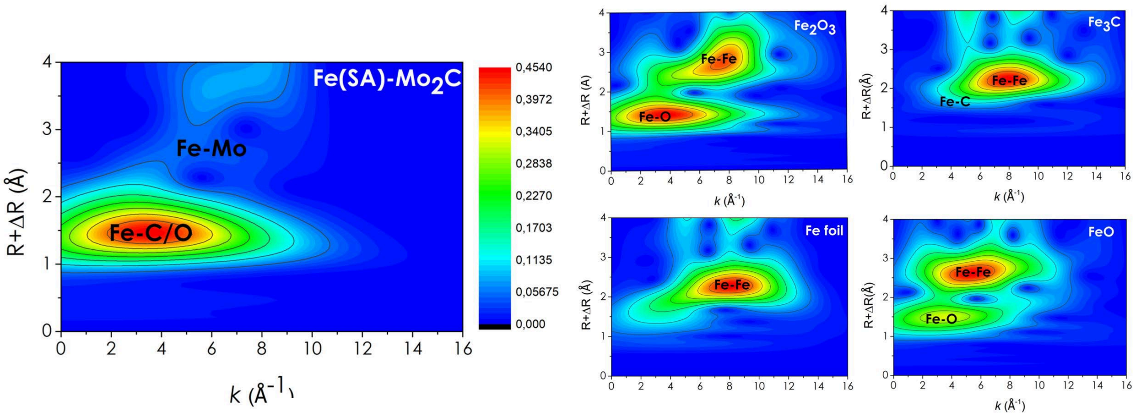

Figure S7. *Wavelet transform plot of the Fe K-edge of the Fe(SA)/Mo_2C and reference materials Fe foil, Fe_3C, FeO, and Fe_2O_3. For Wavelet Transform analysis, the χ(k) exported from Athena was imported into the Hama Fortran code.*[1] *The parameters were listed as follow: R-range, 0.0 - 4.0 Å, k-range, 0 - 16.0 $Å^{-1}$ for sample and Standards; k weight, 2; and Morlet function with κ=8, σ=1 was used as the mother wavelet to provide the overall distribution.*

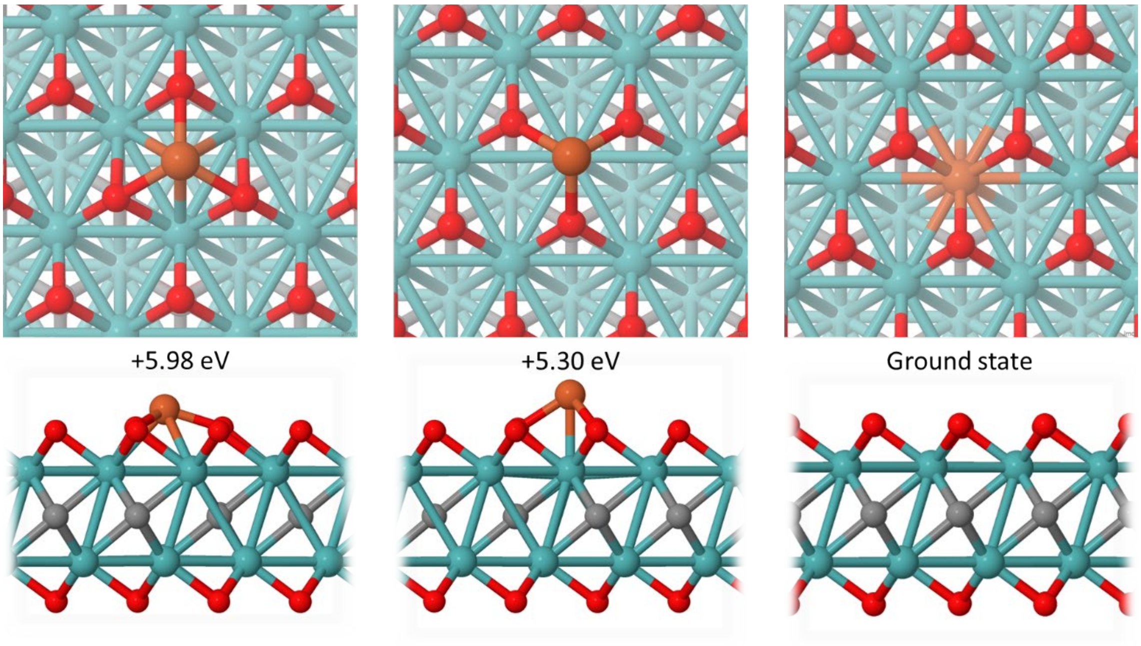

Figure S8. *The three locations that were considered for an Fe atom deposited on the surface of the Mo_2CO_2 MXene model. Frontal and side views are shown on the top and bottom panels, respectively. From left to right, they represent an Fe atom deposited amid the O surface termination over a far Mo site (Fe top Mo far), amid the O surface termination over a near Mo site (Fe top Mo near), or filling an Mo vacancy (Fe sub Mo). The numbers shown under the panels are the energy of the corresponding system, relatively to the ground state (vacancy-confined doping). Colour code: Mo atoms in blue, C in grey, O in red, Fe in orange.*

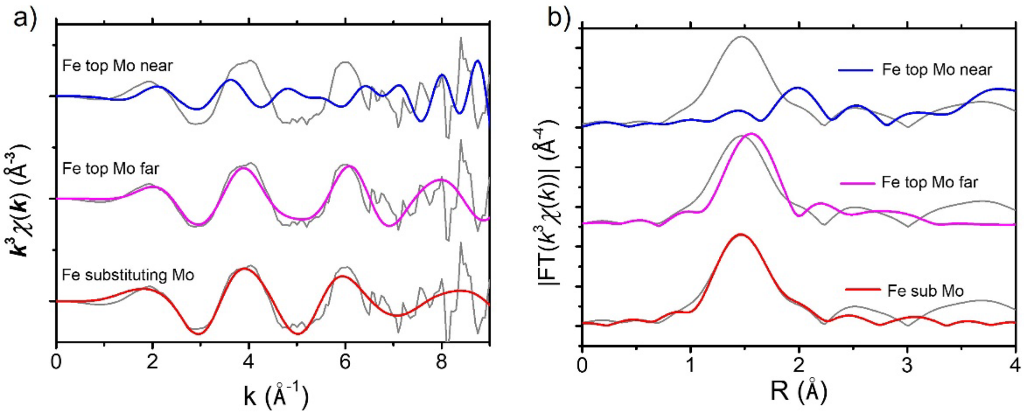

Figure S9. *EXAFS fitting with alternate cif models (see Figure S8) with adsorbed Fe instead of substituted Fe. As can be seen only Fe substituting Mo has a good fit.*

Table S1. *EXAFS Fitting Parameters for Fe(SA)Mo_2C in the 1.0–3.1 Å Range of R-Space and the 2.0–9.0 $Å^{-1}$ Range in k-Space; k-weight = 3. ΔE_0 was set to 0.72 eV, ΔS_0^2 was set to 0.83 both values were chosen after a standard fit of Fe-foil with a Fe-foil cif file.*

Model	Bonds	First Shell N	First Shell R (Å)	σ^2	R factor
**Fe top Mo near**	Fe-O	6±0.23	3.11±0.12	0.021±0.036	0.7687
	Fe-C	1±0.11	4.41±1.44	0.021±0.063	
	Fe-Mo	3±0.27	2.22±0.21	-0.020±0.060	
**Fe top Mo far**	Fe-O	3±0.27	2.07±0.22	0.004±0.004	0.1172
	Fe-Mo	3±0.27	2.30±0.23	0.032±0.027	
**Fe sub Mo**	Fe-O	3±0.27	2.01±0.056	0.002±0.002	0.0172
	Fe-C	3±0.11	2.26±0.14	0.001±0.003	
	Fe-Mo	6±0.26	2.95±0.24	0.045±0.018	

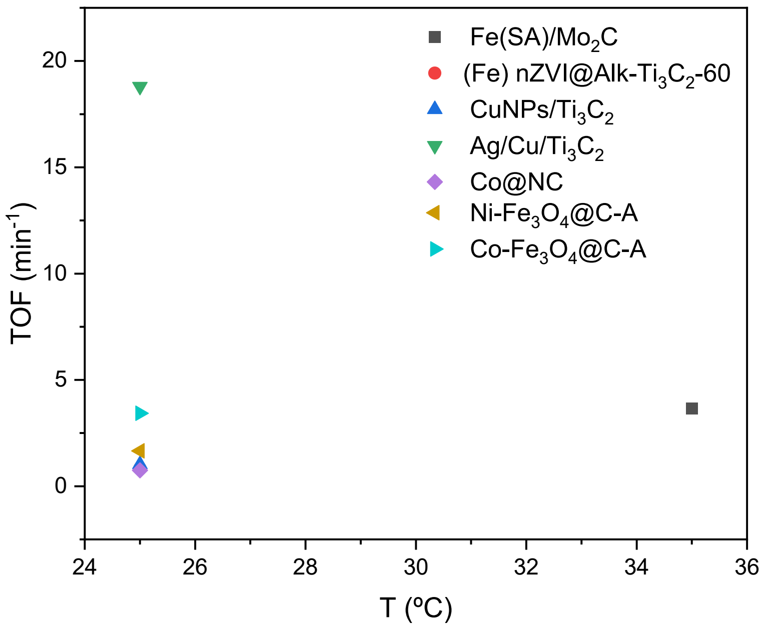

Figure S10. *Comparative TOF values for the reduction of 4-nitrophenol (4-NP) to 4-aminophenol (4-AP) using Fe(SA)/Mo_2C (this work), other MXene catalysts: Ag/Cu/Ti_3C_2,[2] CuNPs/Ti_3C_2,[3] (Fe)nZVI@Alk-Ti_3C_2-60,[4] and other non-precious metal catalyst: Co@NC,[5] Ni-Fe_3O_4@C-A and Co-Fe_3O_4@C-A.[6] Note: Data points labeled as 25 °C were recorded at room temperature.*

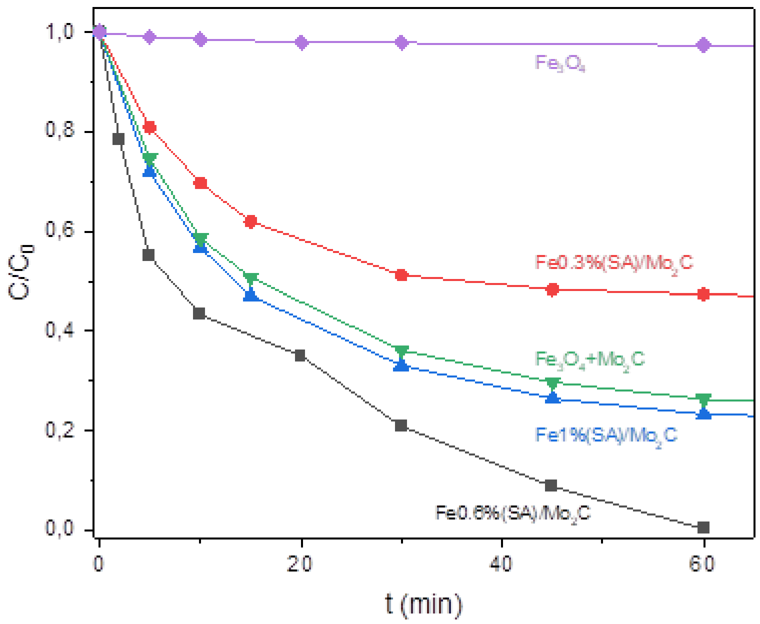

Figure S11. *4-nitrophenol (4-NP) conversion using Fe0,6%(SA)/Mo_2C, Fe1%(SA)/Mo_2C, Fe0,3%(SA)/Mo_2C, a physical mixture of Fe_3O_4 and Mo_2C, and Fe_3O_4 as catalyst.*

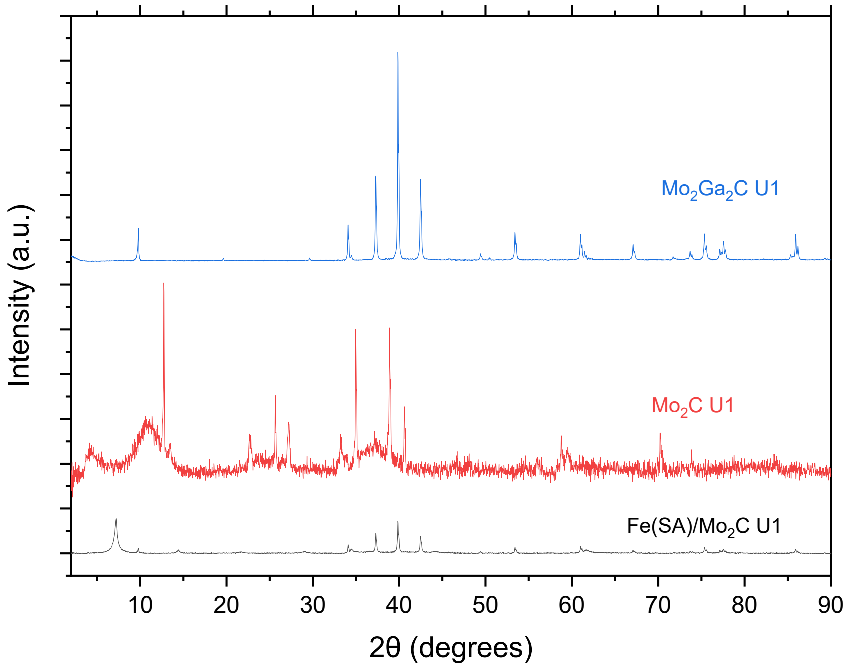

Figure S12. *Comparative XRD of the three materials: Fe(SA)/Mo_2C, Mo_2C and the MAX phase Mo_2Ga_2C after use.*

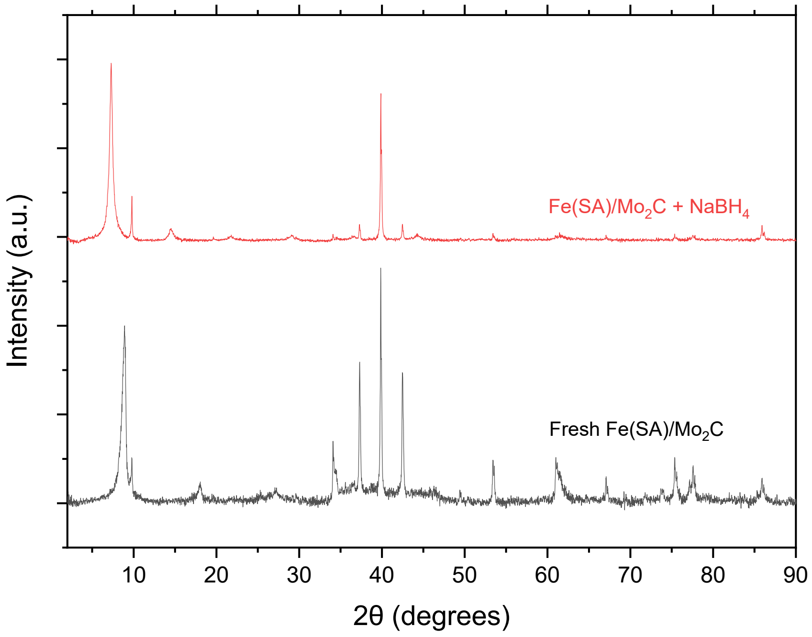

Figure S13. *Comparative XRD analysis of Fresh Fe(SA)/Mo$_2$C catalyst, or in H_2O in presence of $NaBH_4$ in reaction conditions after 60 min.*

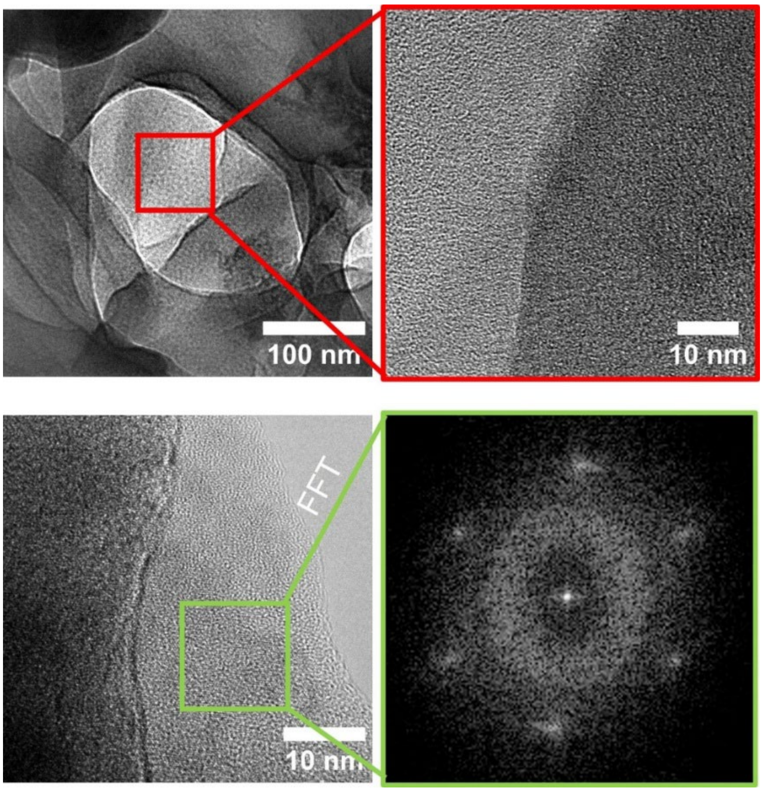

Figure S14. *TEM Images, after 5 reuses. Showing some amorphous regions, and regions intact with the typical Hexagonal shaped FFT of MXene.*

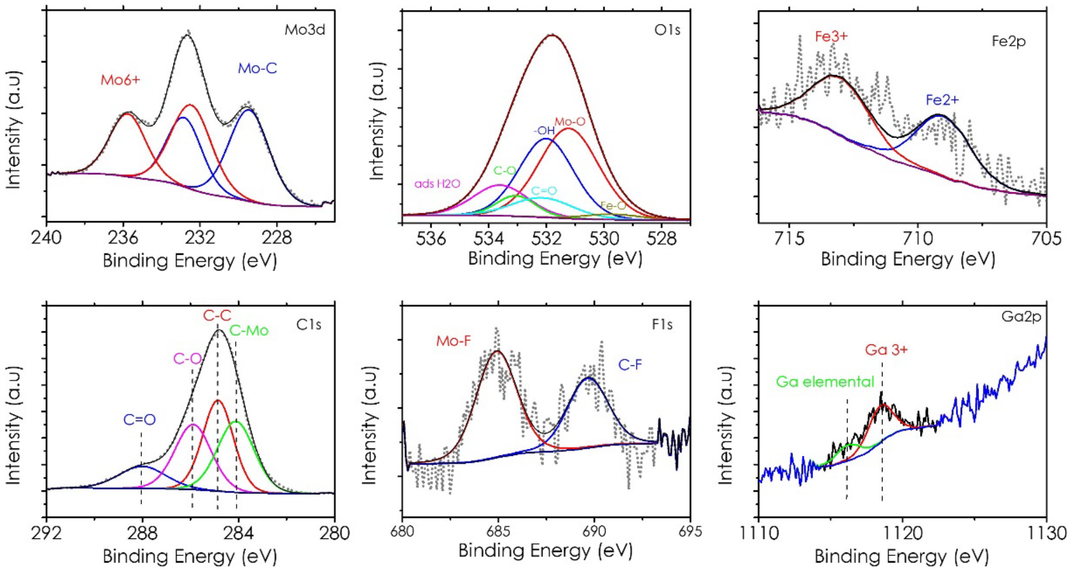

Figure S15. *XPS spectra of Fe(SA)/Mo_2C after five reuses.*

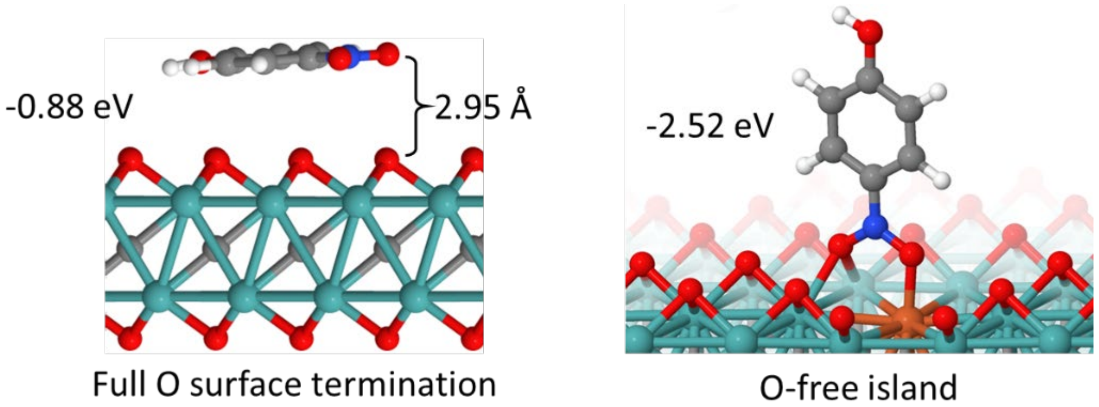

Figure S16. *Adsorption configurations of 4-nitrophenol on two Fe@Mo_2CO_2 MXene models with full O termination (left panel) or with three O vacancies surrounding the Fe atom (right panel). The number in Å is the average distance from the atoms of the adsorbate to the nearest surface O layer, and the numbers in eV are the adsorption energies. Color code: Mo atoms in blue, C in grey, O in red, Fe in orange, N in blue, H in white.*

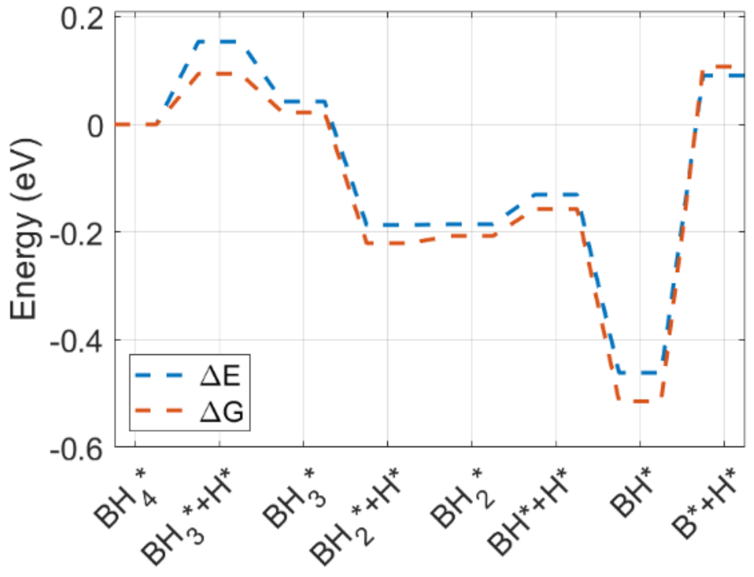

Figure S17. *Comparison between energy (ΔE) and Gibbs free energy (ΔG) for BH_4 dissociation on the Fe@Mo_2CO_2 (black) model of the Fe-doped Mo_2CO_2 MXene surface.*

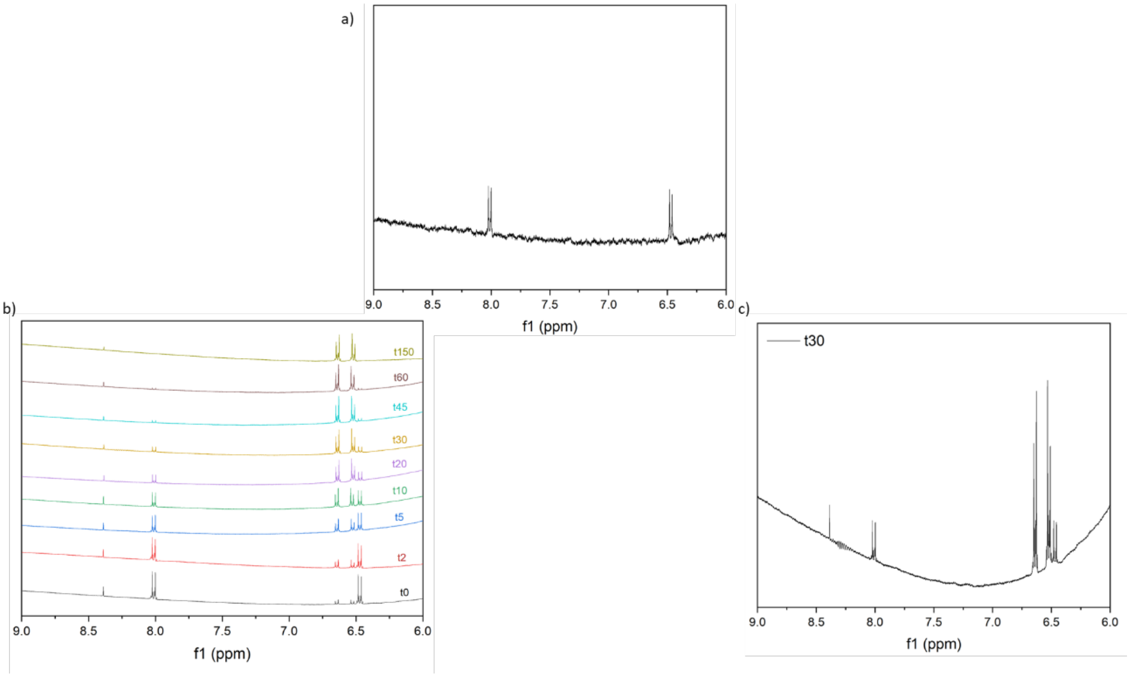

Figure S18. *a) ^{1}H NMR spectra of 4-NP b) ^{1}H NMR spectra recorded during the reduction of 4-nitrophenol (4-NP) to 4-aminophenol (4-AP) catalyzed by Fe(SA)/Mo_2C at different reaction times; samples are labeled as t_x, where x corresponds to the reaction time in minutes. c) Magnified view of the ^{1}H NMR spectrum after 30 min of reaction, showing the absence of detectable intermediate species.*

REFERENCES:

(1) Funke, H.; Scheinost, A. C.; Chukalina, M. Wavelet Analysis of Extended X-Ray Absorption Fine Structure Data. *Phys. Rev. B* **2005**, *71* (9), 094110. https://doi.org/10.1103/PhysRevB.71.094110.

(2) Jiang, P.; Guo, W.; Peng, J.; Peng, W.; Li, H.; Peng, C.; Chen, S. Facile Synthesis of MXene-Supported Bimetallic Ag/Cu Nanoparticles Composite as Efficient Catalyst for 4-Nitrophenol Reduction. *Mater. Lett.* **2023**, *350* (28), 134918. https://doi.org/10.1016/j.matlet.2023.134918.

(3) Liu, L.; Zhao, Q.; Liu, R.; Zhu, L. Hydrogen Adsorption-Induced Catalytic Enhancement over Cu Nanoparticles Immobilized by Layered Ti_3C_2 MXene. *Appl. Catal. B* **2019**, *252* (10), 198–204. https://doi.org/10.1016/j.apcatb.2019.04.026.

(4) Zhang, X.; Sun, H.; Huang, J.; Zheng, Q.; Zhang, F.; Li, H.; Zhang, M.; Zeng, J.; Yan, Z. Alkalized MXene-Supported Nanoscale Zero-Valent Iron in Situ Derived from NH_2-MIL-88B(Fe) for the Highly Efficient Catalytic Reduction of 4-Nitrophenol. *Mater. Today Sustain.* **2022**, *18* (18), 100145. https://doi.org/10.1016/j.mtsust.2022.100145.

(5) Li, X.; Zeng, C.; Jiang, J.; Ai, L. Magnetic Cobalt Nanoparticles Embedded in Hierarchically Porous Nitrogen-Doped Carbon Frameworks for Highly Efficient and Well-Recyclable Catalysis. *J. Mater. Chem. A Mater.* **2016**, *4* (19), 7476–7482. https://doi.org/10.1039/c6ta01054g.

(6) Baye, A. F.; Appiah-Ntiamoah, R.; Kim, H. Synergism of Transition Metal (Co, Ni, Fe, Mn) Nanoparticles and “Active Support” Fe_3O_4@C for Catalytic Reduction of 4-Nitrophenol. *Sci. Total Environ.* **2020**, *712*. https://doi.org/10.1016/j.scitotenv.2019.135492.
